# Intestinal *Cyp24a1* regulates vitamin D locally independent of systemic regulation by renal *Cyp24a1* in mice

**DOI:** 10.1172/JCI179882

**Published:** 2024-12-17

**Authors:** Michaela A.A. Fuchs, Alexander Grabner, Melody Shi, Susan L. Murray, Emily J. Burke, Nejla Latic, Venkataramana Thiriveedi, Jatin Roper, Shintaro Ide, Koki Abe, Hiroki Kitai, Tomokazu Souma, Myles Wolf

**Affiliations:** 1 Division of Nephrology, Department of Medicine, Duke University School of Medicine, Durham, North Carolina, USA.; 2 Division of Nephrology, Department of Medicine and; 3 Hamburg Center for Kidney Health (HCKH), University Medical Center Hamburg-Eppendorf, Hamburg, Germany.; 4 Department of Biomedical Sciences, University of Veterinary Medicine, Vienna, Austria.; 5 Division of Gastroenterology, Department of Medicine and; 6 Duke Clinical Research Institute, Duke University School of Medicine, Durham, North Carolina, USA.; 7 Joan and Sanford I. Weill Department of Medicine, Weill Cornell Medicine, New York, New York, USA.

**Keywords:** Metabolism, Nephrology, Calcium, Chronic kidney disease

## Abstract

Vitamin D regulates mineral homeostasis. The most biologically active form of vitamin D, 1,25-dihydroxyvitamin D (1,25D), is synthesized by CYP27B1 from 25-dihydroxyvitamin D (25D) and is inactivated by CYP24A1. Human monogenic diseases and genome-wide association studies support a critical role for CYP24A1 in regulation of mineral homeostasis, but little is known about its tissue-specific effects. Here, we describe the responses of mice with inducible global deletion, kidney-specific, and intestine-specific deletion of *Cyp24a1* to dietary calcium challenge and chronic kidney disease (CKD). Global and kidney-specific *Cyp24a1* deletion caused similar syndromes of systemic vitamin D intoxication: elevated circulating 1,25D, 25D, and fibroblast growth factor 23 (FGF23), activation of vitamin D target genes in the kidney and intestine, hypercalcemia, and suppressed parathyroid hormone (PTH). In contrast, mice with intestine-specific *Cyp24a1* deletion demonstrated activation of vitamin D target genes exclusively in the intestine, despite no changes in systemic vitamin D levels. In response to a high calcium diet, PTH was suppressed, despite normal serum calcium. In mice with CKD, intestinal *Cyp24a1* deletion decreased PTH and FGF23 without precipitating hypercalcemia. These results implicate kidney CYP24A1 in systemic vitamin D regulation while independent local effects of intestinal CYP24A1 could be targeted to treat secondary hyperparathyroidism in CKD.

## Abstract 2

CYP24A1 regulates vitamin-D effects locally in the intestine, independent of renal systemic vitamin-D control. Intestinal CYP24A1 presents a new target for treatment of secondary hyperparathyroidism.

## Graphical abstract

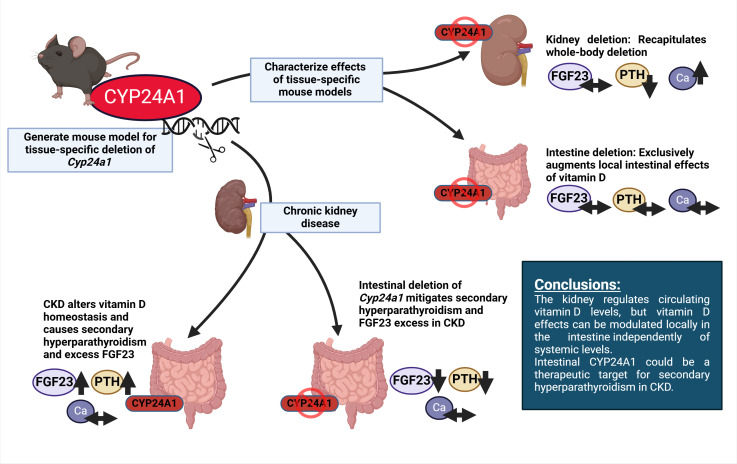

## Introduction

Normal calcium and phosphate homeostasis is essential for myriad cellular and metabolic functions that ensure healthy skeletal development and optimal organ function. Disordered mineral homeostasis can cause metabolic bone diseases and nephrolithiasis and is a common complication of chronic kidney disease (CKD) that contributes to its high rates of cardiovascular disease, fracture, and death (1–7).

Vitamin D, parathyroid hormone (PTH), and fibroblast growth factor 23 (FGF23) regulate calcium and phosphate homeostasis by exerting interconnected effects on the intestine, kidney, parathyroid glands, and bone (8, 9). The biologically active form of vitamin D, 1,25-dihydroxyvitamin D (1,25D; also known as calcitriol), augments intestinal calcium absorption (10, 11). Circulating concentrations of 1,25D are counter regulated by PTH and FGF23: PTH increases and FGF23 decreases 1,25D levels via opposing actions on 1,25D synthesis and degradation (10–12). PTH stimulates (and FGF23 inhibits) the synthetic enzyme CYP27B1 (1α-hydroxylase), which converts 25-hydroxyvitamin D (25D) to 1,25D (13). FGF23 stimulates (and PTH inhibits) the catabolic enzyme CYP24A1 (24-hydroxylase), which inactivates 1,25D to 1,24,25(OH)_3_D and converts 25D to 24,25-dihydroxyvitamin D (24,25D) (14). The products of CYP24A1-catalyzed reactions are eliminated by the liver (14).

Human genetic disorders confirm the physiological importance of CYP24A1. Complete loss-of-function mutations in *CYP24A1* cause 1,25D-mediated hypercalcemia with appropriately suppressed PTH (15, 16). Partial loss-of-function mutations cause calcium nephrolithiasis due to hypercalciuria driven by excessive intestinal calcium absorption that is excreted by the kidney (17). In population-based studies, single nucleotide polymorphisms in or near *CYP24A1* were the only consistent genetic predictors of serum calcium, 25D, PTH, and FGF23 levels (18–21). In a genome-wide association study of FGF23, single nucleotide polymorphisms near *CYP24A1* were associated with higher FGF23 and lower PTH, suggesting that the associated haplotype suppressed CYP24A1 activity, prolonged the half-life of 1,25D, and potentiated its effects (21). CYP24A1 has also been implicated in dysregulation of 1,25D in CKD, which contributes to development of secondary hyperparathyroidism (7, 22–25).

Despite these compelling human data, the role of CYP24A1 in mineral homeostasis remains poorly understood due to insufficient tools to investigate its function in vivo. Most experimental mice with global *Cyp24a1* deletion fail to survive the perinatal period, presumably due to severe hypercalcemia in utero or postnatally (26, 27). A minority manage to survive by markedly reducing their circulating 1,25D levels and avoiding hypercalcemia (26, 27). Despite their low 1,25D levels, surviving *Cyp24a1*-deficient mice manifest excessive end-organ effects of 1,25D, including increased intestinal calcium absorption and suppressed PTH (26, 27). These data suggest important regulatory effects of CYP24A1 locally in the intestine that are independent of circulating 1,25D levels (28), but the lack of conditional mouse models has limited investigation of tissue-specific effects of CYP24A1.

To investigate tissue-specific effects of CYP24A1, we generated new conditional mouse models from which we deleted *Cyp24a1* globally and selectively from the kidney and intestine. Since the kidney is thought to be the primary regulator of circulating 1,25D levels (13, 29, 30), we hypothesized that kidney-specific *Cyp24a1* deletion would replicate the phenotype of global deletion: increased serum 1,25D and vitamin D receptor (VDR) activation in all tissues, including the kidney and intestine, that results in hypercalcemia and suppressed PTH. In contrast, since the intestine is not thought to contribute to circulating 1,25D (30), we hypothesized that intestine-specific *Cyp24a1* deletion would increase VDR activity only in the intestine via local increases in 1,25D activity, and that this would suppress PTH by increasing intestinal calcium absorption, but do so without altering serum 1,25D or calcium levels. Finally, we hypothesized that reducing intestinal CYP24A1 effects would attenuate secondary hyperparathyroidism in CKD.

## Results

### Basal and inducible expression of Cyp24a1 and calcitriol target genes in the kidney and intestine.

**Figure 1:**
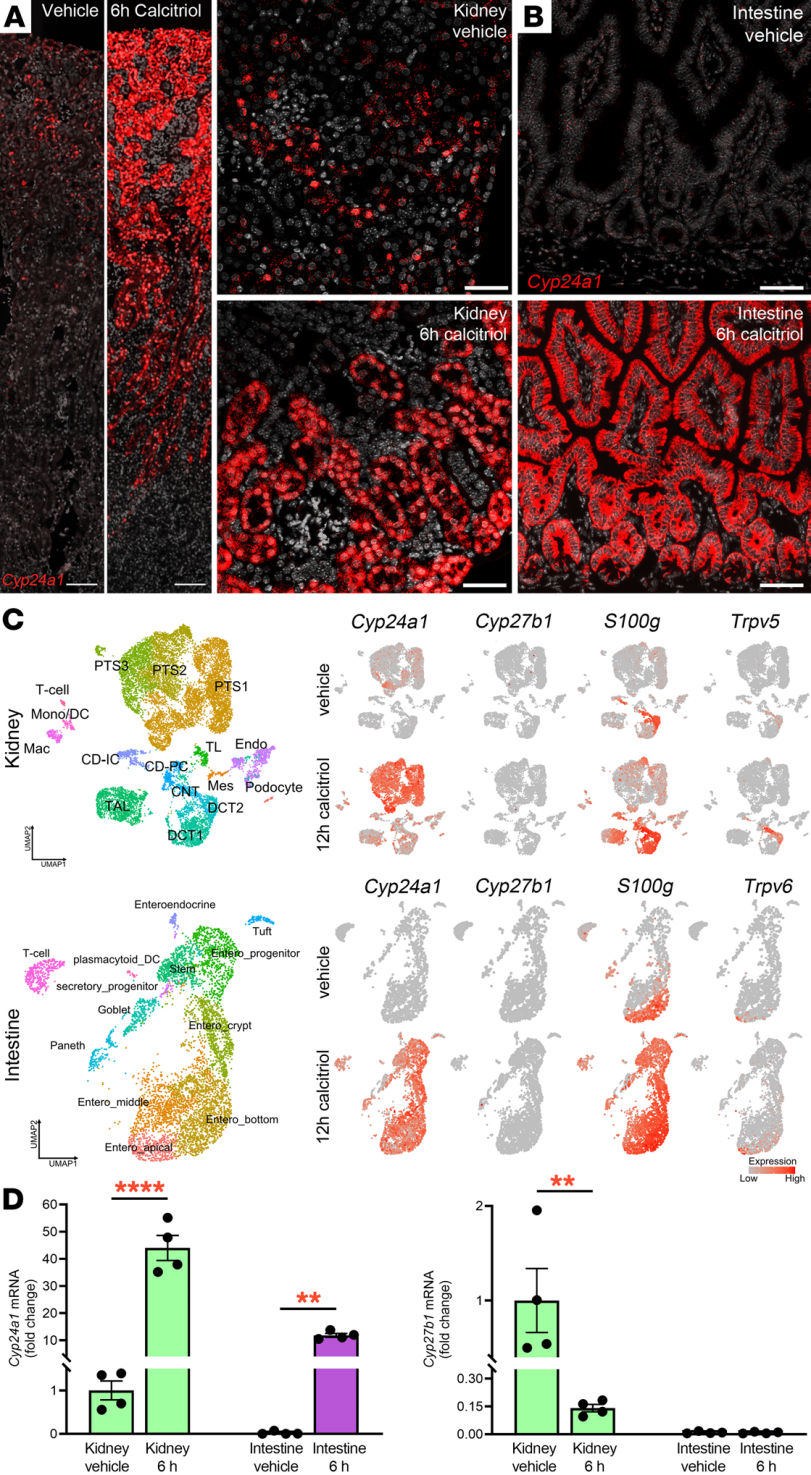
Basal and calcitriol-induced expression of *Cyp24a1* in kidney and small intestine. RNAscope localized expression of *Cyp24a1* in WT mice 6 hours after injection of calcitriol (10 μg/kg) or vehicle. (**A**) In vehicle-treated mice, *Cyp24a1* mRNA expression was localized in proximal tubules near the outer cortex. Calcitriol injection upregulated *Cyp24a1* expression in proximal tubules across the cortex (scale bars: 100 μm, low power images; 50 μm, high power images). (**B**) Expression of *Cyp24a1* was extremely low in the small intestines of vehicle-treated mice and was upregulated in most epithelial cells by calcitriol (scale bars: 50 μm). (**C**) Integrated single cell transcriptome map of kidney and duodenal epithelial cell responses to calcitriol. Unsupervised clustering identified all major cell types. *Cyp24a1* was basally expressed in kidney proximal tubules and strongly upregulated by calcitriol. Calcitriol variably stimulated expression of *S100g* and *Trpv5* in the kidney. In the intestine, basal *Cyp24a1* expression was minimal, but was strongly upregulated by calcitriol in all enterocyte populations. Intestinal *Cyp27b1* expression was virtually undetectable at baseline and after calcitriol injection. Calcitriol strongly stimulated expression of *S100g* from the bottom of intestinal villi to all enterocytes and stimulated *Trpv6* expression mostly in apical villi. (**D**) Quantitative polymerase chain reaction confirmed the expression patterns of *Cyp24a1* and *Cyp27b1* in kidney and intestine 6 hours (h) after calcitriol injection. Representative images from ≥ 4 animals. *n* ≥ 4 for qPCR. Results are mean ± SEM. Statistical significance was calculated by 2-tailed Student’s *t* test; ^**^P < 0.005; ^****^P < 0.0001. Total numbers of cells recovered for single cell RNA sequencing analyses are, for the kidney: 14,169 cells; intestine: 6,957 cells. PT, proximal tubule (S1, S2, S3 segments); TL, thin limb; TAL, thick ascending limb; DCT, distal convoluted tubule; CNT, connecting tubule; CD, collecting duct; PC, principal cells; IC, intercalated cells; Mes, mesangial cells; Endo, endothelial cells; Mac, macrophages; Mono/DC, monocytes and dendritic cells; Neut, neutrophils; Entero, enterocytes.

Calcitriol is a potent inducer of CYP24A1 (13, 29, 30). We used RNAscope technology to visualize Cyp24a1 mRNA expression in the kidney and intestine after injecting 12-week-old WT mice with calcitriol (10 μg/kg body weight) or vehicle. In the kidney, Cyp24a1 was basally expressed in proximal tubules of the outer cortex (Figure 1A). By 6 hours after calcitriol injection, Cyp24a1 mRNA expression markedly increased and expanded across the cortex along all segments of the proximal tubule (Figure 1A). Basal expression of Cyp24a1 was much lower in the small intestine than in the kidneys but was also highly stimulated in epithelial cells of villi and crypts by 6 hours after calcitriol injection (Figure 1B).

To map the cell-specific response to calcitriol, we employed single-cell RNA sequencing (scRNA-seq) in WT mice. Our methodology yielded high cell viability (> 90%) and a minimal number of doublets. Analysis of scRNA-seq data confirmed basal expression of *Cyp24a1* in the kidney’s proximal tubule but not the small intestine and marked induction of *Cyp24a1* expression in both proximal tubular and duodenal epithelial cells in response to calcitriol injection (Figure 1C). Basal expression of *Cyp27b1* (encodes 1α-hydroxylase) was low in the kidney and intestine and unchanged by calcitriol injection (Figure 1C). Using quantitative polymerase chain reaction (qPCR), we confirmed the basal and calcitriol-inducible expression patterns of *Cyp24a1* in the kidney and intestine (Figure 1D) and found that the low basal expression of *Cyp27b1* in the kidney was further suppressed by calcitriol, consistent with known negative feedback loops (31).

High basal expression of *Vdr*, which encodes the VDR, was mostly unchanged in response to calcitriol in both kidney and intestine (Supplemental Figure 1; supplemental material available online with this article; https://doi.org/10.1172/JCI179882DS1). Downstream of the VDR, scRNA-seq analysis of the kidney showed basal expression of *S100g*, which encodes calbindin D9K, a cytosolic calcium-binding protein involved in calcium transport (32), in the distal tubule and connecting tubules. Expression of *S100g* increased and expanded in response to calcitriol injection to the thick ascending limb of Henle, proximal tubule, and endothelial cells (Figure 1C). In the intestine, *S100g* expression was strongly recruited in all enterocytes above its more localized basal expression (Figure 1C). Expression of organ-specific apical calcium channels was restricted to specialized cell populations. Kidney expression of *Trpv5*, which encodes transient receptor potential cation channel subfamily V member 5 (33), was expressed most prominently in the connecting and distal tubule, whereas *Trpv6* was mostly expressed in apical sections of intestinal villi (Figure 1C). Based on these scRNA-seq data, we utilized *S100g*, *Trpv5*, and *Trpv6* expression as molecular indicators of 1,25D-VDR activation in subsequent experiments.

### Genetic strategy to study tissue-specific effects of Cyp24a1 in adult mice.

**Figure 2:**
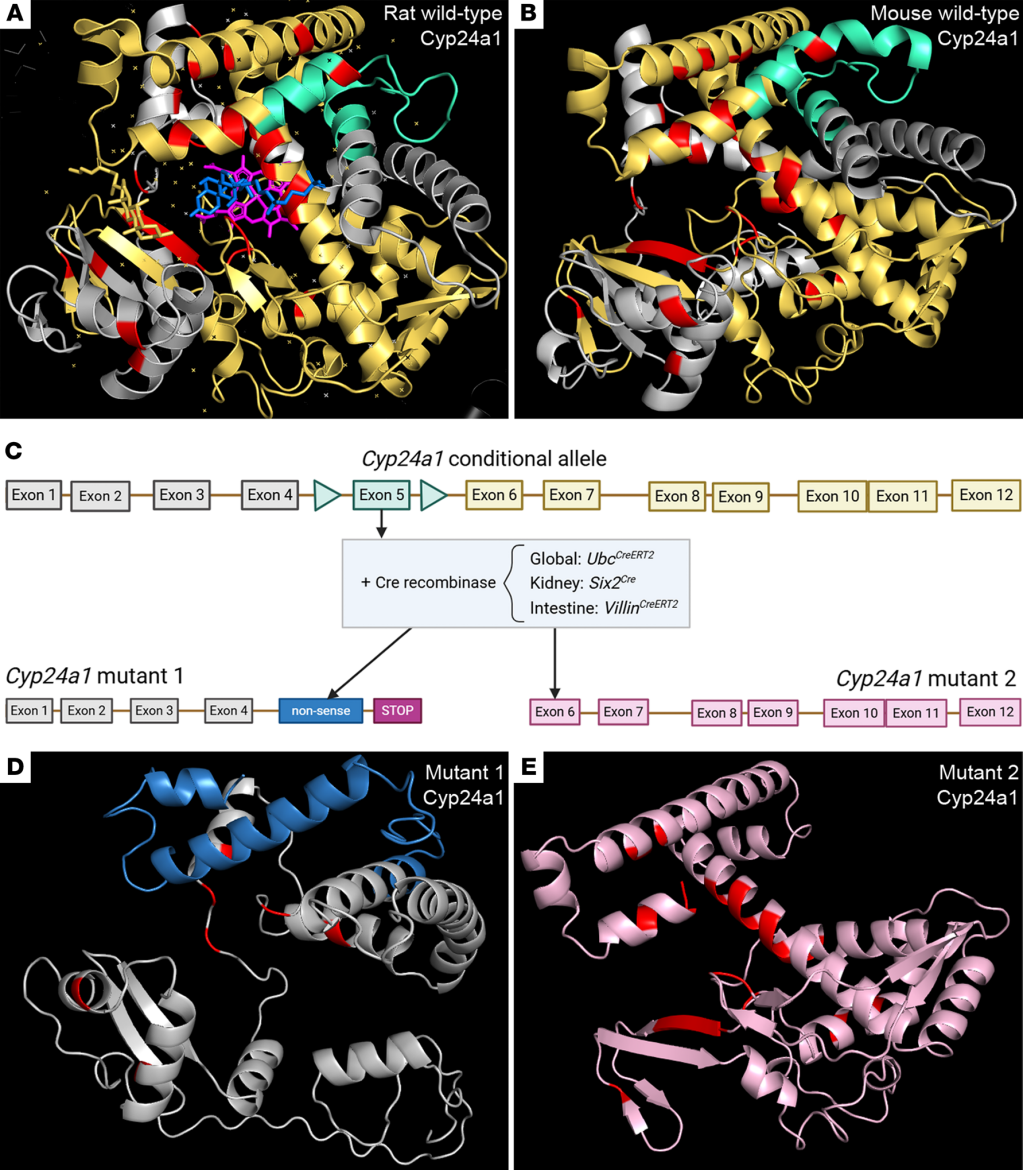
In silico modeling of effects of excising exon 5 on Cyp24a1 protein structure. (**A**) Previously published crystal structure of rat Cyp24a1. The heme group at the catalytic center is shown in bright pink with a solvent molecule of the crystallization buffer in blue occupying the substrate channel. Parts of the α-helices E and F encoded by exon 5 are highlighted in turquoise; these form part of the substrate channel near the catalytic center. Conserved structures before exon 5 are shown in pale grey and domains predicted to be disrupted by excision of exon 5 are shown in yellow. Amino acids important for enzymatic function or reported in pathological human mutations are shown in red (Supplemental Table 1). (**B**) Predicted structure of mouse WT Cyp24a1, which shares 94.75% homology to rat Cyp24a1, color-coded as in the rat protein structure. (**C**) Schema of genetic strategy to delete exon 5 globally and from the kidney and intestine. (**D**) In silico modeling of mutant protein 1 revealed a heavily truncated protein due to a frameshift leading to a different amino acid sequence downstream of the deletion site (blue) and introduction of a premature stop codon. (**E**) In silico modeling of mutant protein 2, which might be produced from a new open reading frame at the site of exon 5 deletion, consists of domains encoded by exons 6–12 of native *Cyp24a1* (pink). This candidate peptide would lack the mitochondrial membrane insertion sites of WT Cyp24a1 and is unlikely to be trafficked into the mitochondria, which is required for normal protein function.

We used CRISPR/Cas9 technology to flank exon 5 of *Cyp24a1* with loxP sites. Exon 5 encodes parts of the E and F α-helices that are essential components of the substrate channel of CYP24A1 (34). To predict the effects of deleting exon 5 on protein structure, we modeled WT mouse Cyp24a1, which is highly homologous to rat Cyp24a1, the crystal structure of which is published (Figure 2, A and B) (34). Many human loss-of-function mutations of CYP24A1 are precipitated by mutations in single nucleotides (15). We therefore included amino acids essential for enzymatic function and pathological mutation sites in the analysis (Figure 2, A and B; important amino acids highlighted in red, and Supplemental Table 1). Modeling the potential new peptide sequences of murine Cyp24a1 following excision of exon 5 by Cre-recombination suggested 2 open reading frames that could lead to mutant proteins of more than 200 residues (Figure 2C). Mutant protein 1 would utilize the native transcription start, but deletion of exon 5 would cause a downstream frame shift that modifies the amino acid sequence in functionally important domains and would generate a premature stop codon that should render the mutant mRNA a target for nonsense-mediated decay (Figure 2D, blue, and Supplemental Table 1). In addition, the truncated mutant protein 1 would lack exons 9–12, which are essential for binding of the heme group at the catalytic center of CYP24A1, and it would also lack the binding site for adrenodoxin, which is the essential electron transfer partner needed for normal enzymatic function (Supplemental Table 1) (34, 35). The second open reading frame would yield a protein that lacks the initial half of the WT CYP24A1 protein (Figure 2E; pink). The truncated mutant protein 2 would lack residues essential for forming the substrate channel and substrate binding capability (L129, I131, and W134), leading to changes in the geometry of the enzymatic catalytic center (34, 35). Furthermore, the N-terminus missing from mutant protein 2 includes the mitochondrial membrane insertion sequences (34, 35), which are essential for transporting the protein into mitochondria (36). Together, these models suggest that excision of exon 5 would eliminate biological activity of Cyp24a1.

To generate experimental mice for investigation of tissue-specific effects of *Cyp24a1*, we bred homozygous carriers for the floxed allele of *Cyp24a1* (*Cyp24^fl/fl^*) and crossed them with 3 Cre-recombinase lines. To generate mice with inducible global *Cyp24a1* deletion, we crossed tamoxifen-inducible *Ubc-CreERT2* mice with *Cyp24^fl/fl^* mice (*Ubc^CreERT2^Cyp24^fl/fl^*) (37). To induce kidney-specific *Cyp24a1* deficiency, we crossed *Six2-TGC^tg^* mice with *Cyp24^fl/fl^* mice (*Six2^Cre^Cyp24^fl/fl^*); *Six2-TGC^tg^* enables deletion of target genes from the nephron without affecting other extrarenal sites, such as the intestine (38, 39). To establish a model of intestine-specific *Cyp24a1* deficiency, we crossed tamoxifen-inducible *Villin-CreERT2* mice with *Cyp24^fl/fl^* mice (*Villin^CreERT2^Cyp24^fl/fl^*). The *Villin^CreERT2^* transgene specifically targets intestinal epithelial cells, but not other tissues that express *Villin1*, including the kidneys (40).

**Figure 3:**
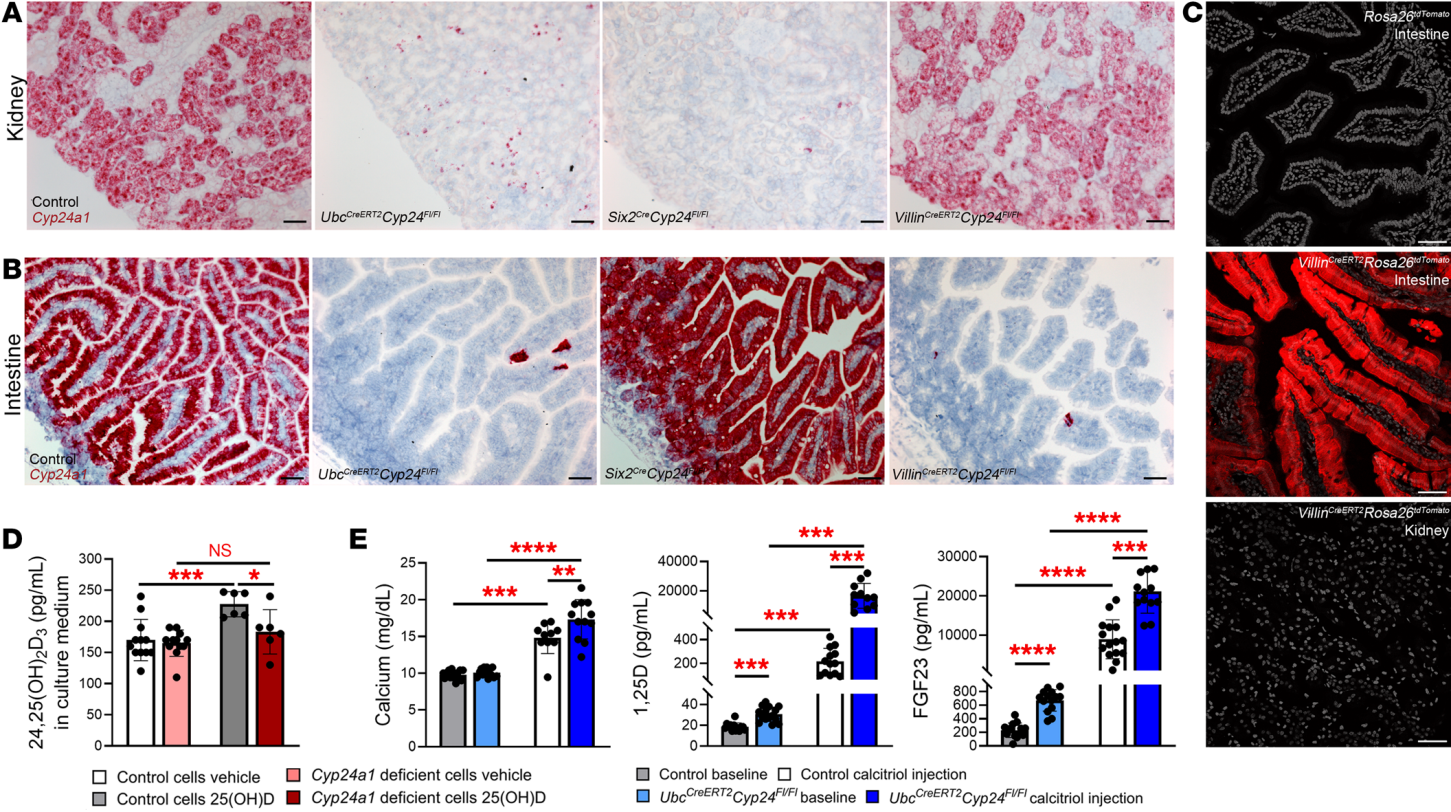
Validation of tissue specificity and loss of function after *Cyp24a1* deletion. Homozygous *Cyp24^fl/fl^* mice were crossed with different Cre-drivers to induce global (*Ubc-CreERT2*), kidney-specific (*Six2-Cre*), or intestine-specific (*Villin-CreERT2*) deletion of *Cyp24a1*; Cre-negative littermates served as controls. Recombination efficiency was evaluated 6 hours after calcitriol injection, 10 μg/kg. (**A**) In situ hybridization using a probe specific to exon 5 of *Cyp24a1* confirmed successful deletion from most kidney cells in *Ubc^CreERT2^Cyp24^fl/fl^* and *Six2^Cre^Cyp24^fl/fl^* mice, but not *Villin^CreERT2^Cyp24^fl/fl^* mice. (**B**) Evaluation of the small intestines confirmed successful deletion of *Cyp24a1* in *Ubc^CreERT2^Cyp24^fl/fl^* and *Villin^CreERT2^Cyp24^fl/fl^* mice, but not *Six2^Cre^Cyp24^fl/fl^* mice. (**C**) Kidneys of *Rosa26^tdTomato^* controls and *Villin^CreERT2^Rosa26^tdTomato^* mice demonstrated no red fluorescence after tamoxifen treatment, while intestinal epithelia showed robust red tdTomato fluorescence, confirming intestinal specificity of *Villin-CreERT2*. (**D**) Primary cultures of proximal tubule cells derived from kidney-specific *Six2^Cre^Cyp24^fl/fl^* mice and controls were used to evaluate Cyp24a1 function in vitro. Levels of 24,25D increased significantly in cultures of WT cells after 12 hours of treatment with 7nM 25D (grey), whereas there was no change in 24,25D levels in cultures of proximal tubule cells derived from *Six2^Cre^Cyp24^fl/fl^* mice (red). (**E**) *Ubc^CreERT2^Cyp24^fl/fl^* mice received 2 calcitriol injections, 2.5 μg/kg, 48 hours apart 1 week after the last tamoxifen injection. At baseline, *Ubc^CreERT2^Cyp24^fl/fl^* mice had higher 1,25D and FGF23 levels, consistent with impaired Cyp24a1 function. Calcitriol injection increased serum calcium, 1,25D, and FGF23 levels in all mice, but exacerbated increases in *Ubc^CreERT2^Cyp24^fl/fl^* mice versus controls, indicating failure of these mice to metabolize exogenous calcitriol. *n* ≥ 4 for all measurements. Results are mean ± SEM. Statistical significance was calculated using mixed effect analysis with Holm-Šidák’s correction. ^*^P < 0.05; ^**^P < 0.005; ^***^P < 0.0005; ^****^P < 0.0001. Representative images from ≥ 4 animals of each strain that were evaluated. Scale bars: 50 μm.

To analyze the efficiency and specificity of Cre-mediated *Cyp24a1* deletion at the cellular level, we designed an in situ hybridization probe specific to the 92 bp sequence of floxed exon 5; detection of the probe would identify cells that escaped genomic recombination. Six hours after injecting calcitriol 10 μg/kg to stimulate *Cyp24a1* mRNA expression, in situ hybridization demonstrated minimal nonrecombined *Cyp24a1* mRNA in the kidneys of *Ubc^CreERT2^Cyp24^fl/fl^* and *Six2^Cre^Cyp24^fl/fl^* mice compared with controls, but intact kidney expression of *Cyp24a1* mRNA in *Villin^CreERT2^Cyp24^fl/fl^* mice (Figure 3A). Analyses of the small intestines demonstrated minimal residual *Cyp24a1* mRNA expression in the intestine of *Ubc^CreERT2^Cyp24^fl/fl^* and *Villin^CreERT2^Cyp24^fl/fl^* mice compared with controls, but intact expression in the intestine of *Six2^Cre^Cyp24^fl/fl^* mice (Figure 3B). Since *Villin1* is endogenously expressed in adult proximal tubule (41), we further tested the specificity of the *Villin^CreERT2^* transgene by generating a *Villin^CreERT2^Rosa26^tdTomato^* mouse line. We observed robust recombination marked by tdTomato expression in intestinal epithelial cells, but no recombination in the kidney after tamoxifen treatment (Figure 3C), further confirming the specificity of intestinal *Cyp24a1* deletion in *Villin^CreERT2^Cyp24^fl/fl^* mice.

### Functional validation of the genetic approach to Cyp24a1 deletion in mice.

To verify the results of the in silico modeling of protein structure, we assessed the biological effect of excising exon 5 of *Cyp24a1* in vitro and in vivo. First, we designed an in vitro system of primary proximal tubule cells derived from *Six2^Cre^Cyp24^fl/fl^* mice and their respective Cre-negative littermates. We treated the cultured cells with 25D, 7 nM, or vehicle for 12 hours and measured 24,25D concentrations in the media by liquid chromatography-tandem mass spectrometry (LC-MS). Concentrations of 24,25D increased significantly in the media of proximal tubular cells with intact *Cyp24a1*, but were unchanged in the media of *Cyp24a1-*deficient cells (Figure 3D). These data confirm absence of Cyp24a1 function in cells that underwent Cre-loxP recombination.

Next, we tested the biological effect of excising exon 5 of *Cyp24a1* in vivo by assessing the ability of *Ubc^CreERT2^Cyp24^fl/fl^* mice to metabolize exogenous calcitriol, which global constitutive *Cyp24a1*–deficient mice are unable to do (27, 42). We injected *Ubc^CreERT2^Cyp24^fl/fl^* mice with 2 doses of calcitriol, 2.5 μg/kg, 48 hours apart and collected blood at baseline and 24 hours after the last calcitriol injection. Calcitriol injection increased serum calcium, 1,25D and FGF23 levels in control mice but precipitated more severe increases in each of these parameters in *Ubc^CreERT2^Cyp24^fl/fl^* mice (Figure 3E). These data demonstrate that excision of exon 5 prevents mice from inactivating exogenous calcitriol as seen in previous reports of constitutive global knock-out mice. In aggregate, these validation data support our use of the *Ubc^CreERT2^Cyp24^fl/fl^*, *Six2^Cre^Cyp24^fl/fl^*, and *Villin^CreERT2^Cyp24^fl/fl^* mice to study tissue-specific effects of Cyp24a1.

### Global and kidney-specific deletion of Cyp24a1 increase systemic effects of vitamin D.

**Figure 4:**
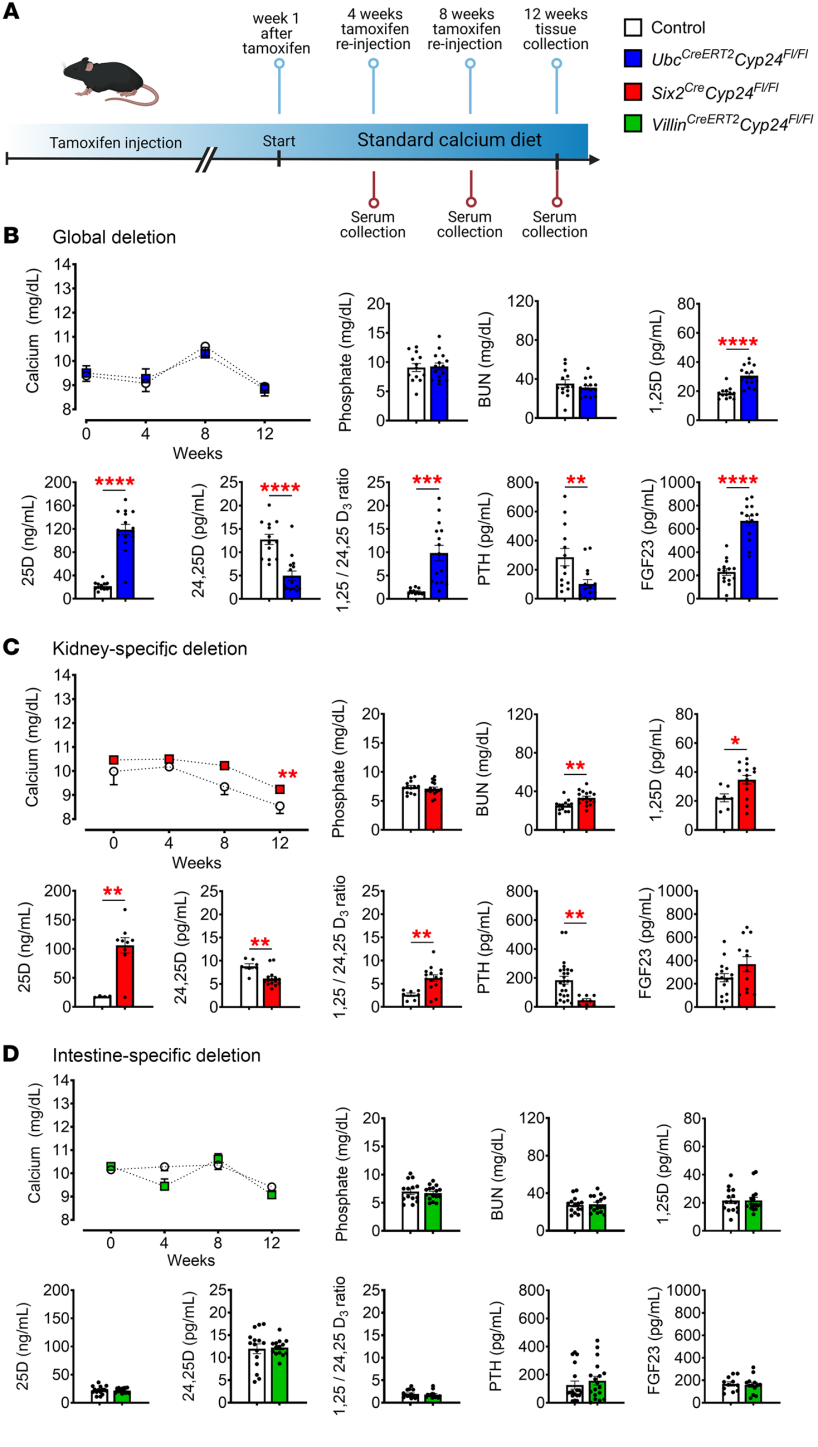
Serologic markers of mineral homeostasis in response to standard calcium diet. (**A**) Schema indicates the protocol of tamoxifen injection (i.p.) and experimental timeline. Deletion of *Cyp24a1* was induced at 12 weeks of age and the observation period began 1 week after the last injection in *Ubc^CreERT2^Cyp24^fl/fl^* and *Villin^CreERT2^Cyp24^fl/fl^* mice. *Six2^Cre^Cyp24^fl/fl^* mice were enrolled into the study at 13 weeks of age. Littermates without Cre-recombinase served as controls and received the same treatments. Serological results for (**B**) *Ubc^CreERT2^Cyp24^fl/fl^* mice and (**C**) *Six2^Cre^Cyp24^fl/fl^* mice demonstrate evidence of impaired systemic Cyp24a1 effects, including high 25D and 1,25D, low 24,25D, a high 1,25D:24,25D ratio, and excessive systemic 1,25D effects marked by high FGF23 and suppressed PTH. (**D**) *Villin^CreERT2^Cyp24^fl/fl^* mice demonstrate no changes in any of these parameters. *n* ≥ 4 for all measurements. Results are mean ± SEM. Statistical significance was calculated by mixed effect analysis with Holm-Šidák’s correction and 2-tailed Student’s *t* test. ^*^P < 0.05; ^**^P < 0.005; ^***^P < 0.0005; ^****^P < 0.0001.

To investigate the physiological effects of *Cyp24a1* deletion, we evaluated adult mice that consumed a standard diet containing 0.8% calcium over a 12-week period after induction with tamoxifen, which began at 12 weeks of age (Figure 4A). Throughout the observation period, *Ubc^CreERT2^Cyp24^fl/fl^* mice demonstrated no significant changes in serum calcium, phosphate, or blood urea nitrogen (BUN) compared with control animals, but serum 1,25D and 25D levels increased in association with suppressed PTH and elevated FGF23, consistent with excess systemic effects of 1,25D (Figure 4B). Decreased 24,25D levels and increased 1,25D: 24,25D ratio confirm that decreased systemic Cyp24a1 activity caused the increases in systemic 1,25D levels (Figure 4B).

Kidney-specific deletion of *Cyp24a1* in *Six2^Cre^Cyp24^fl/fl^* mice resulted in mild hypercalcemia, but serum phosphate levels were unchanged (Figure 4C). BUN was modestly elevated (Figure 4C), but serum creatinine was unchanged in *Six2^Cre^Cyp24^fl/fl^* mice versus controls (data not shown), suggesting prerenal azotemia due to mild hypercalcemia, as has been reported in other mouse models of hypercalcemia (43). Kidney histology confirmed no evidence of glomerular or tubular damage (Supplemental Figure 2A). Like *Ubc^CreERT2^Cyp24^fl/fl^* mice, *Six2^Cre^Cyp24^fl/fl^* mice also demonstrated decreased 24,25D levels, increased 1,25D, 25D, and 1,25D:24,25D ratio, and decreased PTH. The similar serum vitamin D profiles of *Ubc^CreERT2^Cyp24^fl/fl^* and *Six2^Cre^Cyp24^fl/fl^* mice suggest that kidney deletion of *Cyp24a1* is sufficient to replicate the effects of global *Cyp24a1* deletion on systemic vitamin D levels. In contrast to the *Ubc^CreERT2^Cyp24^fl/fl^* and *Six2^Cre^Cyp24^fl/fl^* mice, *Villin^CreERT2^Cyp24^fl/fl^* mice fed a standard calcium diet showed no changes in serum vitamin D levels or any other biochemical measure of mineral homeostasis (Figure 4D).

### Molecular effects of tissue-specific Cyp24a1 deletion.

**Figure 5:**
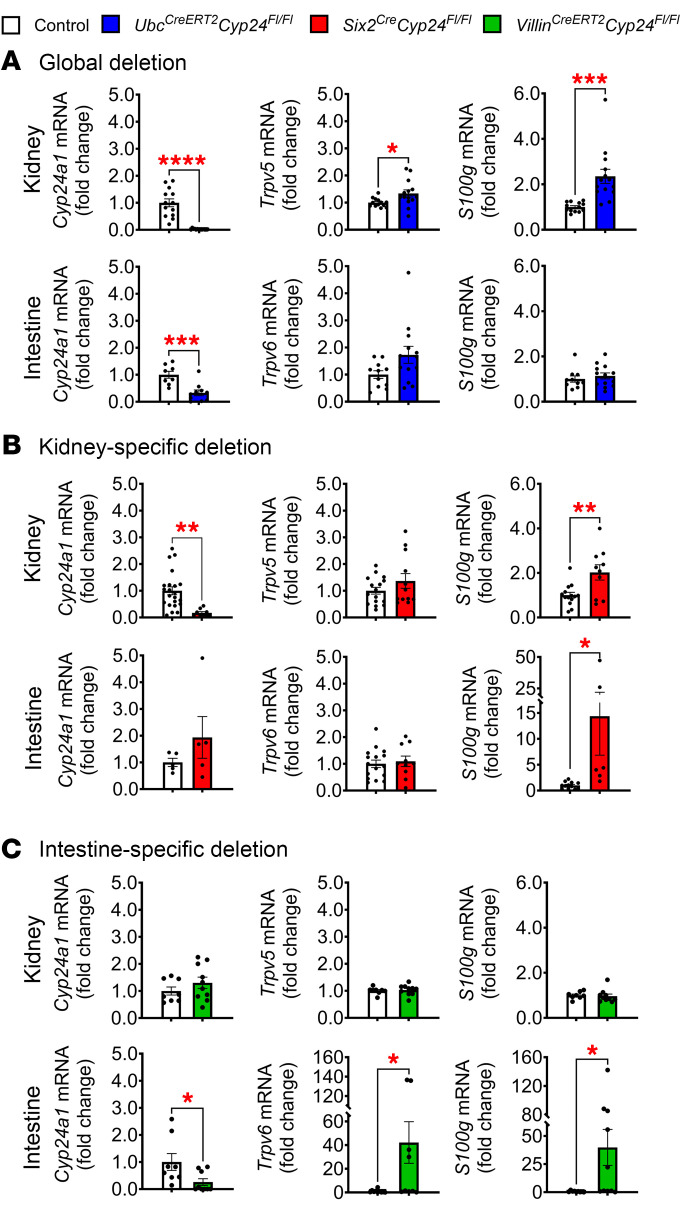
*Cyp24a1* and VDR target gene expression in response to standard calcium diet. Expression levels of *Cyp24a1* and VDR target genes in response to standard calcium diet in the kidney and intestine of (**A**) *Ubc^CreERT2^Cyp24^fl/fl^*, (**B**) *Six2^Cre^Cyp24^fl/fl^*, and (**C**) *Villin^CreERT2^Cyp24^fl/fl^* mice confirm *Cyp24a1* deletion from the kidney and intestine of *Ubc^CreERT2^Cyp24^fl/fl^* mice, from just the kidney of *Six2^Cre^Cyp24^fl/fl^* mice, and from just the intestine in *Villin^CreERT2^Cyp24^fl/fl^* mice. Select VDR target genes were upregulated in the kidneys and intestines of *Ubc^CreERT2^Cyp24^fl/fl^* and *Six2^Cre^Cyp24^fl/fl^* mice, whereas VDR target genes were upregulated only in the intestine of *Villin^Cre^Cyp24^fl/fl^* mice. *n* ≥ 4 for all measurements. Results are mean ± SEM. Statistical significance was calculated by 2-tailed Student’s *t* test. ^*^P < 0.05; ^**^P < 0.005; ^***^P < 0.0005; ^****^P < 0.0001.

To investigate the molecular effects of *Cyp24a1* deletion, we used qPCR to measure mRNA expression of the VDR target genes in the kidney and intestine that were suggested by the scRNA-seq data (Figure 1C). Consistent with the in situ hybridization results (Figure 3, A and B), both *Ubc^CreERT2^Cyp24^fl/fl^* and *Six2^Cre^Cyp24^fl/fl^* mice showed markedly reduced expression of *Cyp24a1* in the kidney when using a TaqMan probe targeting exons 5 and 6; intestinal *Cyp24a1* expression was also markedly reduced in the global *Ubc^CreERT2^Cyp24^fl/fl^* mice, but not in *Six2^Cre^Cyp24^fl/fl^* mice (Figure 5, A and B). Consistent with their increased systemic 1,25D levels, several VDR target genes were significantly upregulated in the kidneys and intestines of *Ubc^CreERT2^Cyp24^fl/fl^* and *Six2^Cre^Cyp24^fl/fl^* mice versus controls (Figure 5, A and B). Given their higher 1,25D and reduced PTH levels, kidney expression of *Cyp27b1* mRNA was appropriately suppressed in both *Ubc^CreERT2^Cyp24^fl/fl^* and *Six2^Cre^Cyp24^fl/fl^* mice (data not shown).

*Villin^CreERT2^Cyp24^fl/fl^* mice demonstrated reduced intestinal but intact kidney expression of *Cyp24a1*, confirming successful tissue-specific deletion, as indicated by in situ hybridization and the *Rosa26^tdTomato^* reporter mice (Figure 3, B and C). In contrast to *Ubc^CreERT2^Cyp24^fl/fl^* and *Six2^Cre^Cyp24^fl/fl^*, *Villin^CreERT2^Cyp24^FL/Fl^* mice showed no changes in expression levels of any VDR target genes in the kidney, whereas expression of intestinal VDR target genes was significantly upregulated (Figure 5C). Collectively, these data suggest that intestinal deletion of *Cyp24a1* increased local 1,25D effects in the intestine, independent of changes in systemic vitamin D levels. Interestingly, these molecular effects did not result in changes in systemic mineral homeostasis, perhaps because of the modest dietary calcium load.

### High calcium diet reveals the physiological effects of intestine-specific deletion of Cyp24a1.

**Figure 6:**
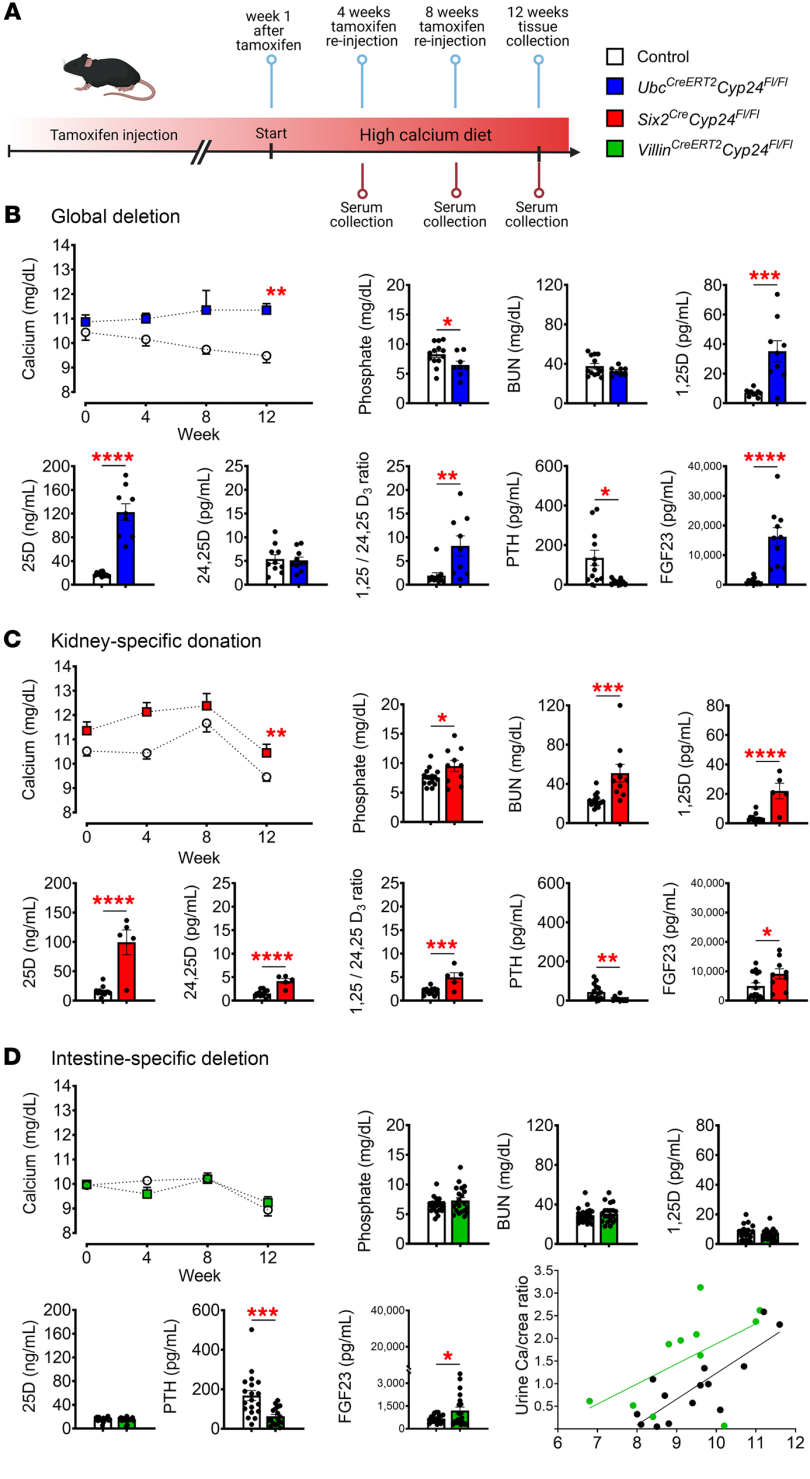
Serologic markers of mineral homeostasis in response to high calcium diet. (**A**) Schema indicates the study protocol of tamoxifen injection (i.p.) and experimental timeline. Deletion of *Cyp24a1* was induced at 12 weeks of age and the observation period and diet change began 1 week after the last injection in *Ubc^CreERT2^Cyp24^fl/fl^* and *Villin^CreERT2^Cyp24^fl/fl^* mice. *Six2^Cre^Cyp24^fl/fl^* mice were enrolled into the study at 13 weeks of age. Littermates without Cre recombinase served as controls and received the same treatments. Serological results for (**B**) *Ubc^CreERT2^Cyp24^fl/fl^* and (**C**) *Six2^Cre^Cyp24^fl/fl^* mice demonstrate hypercalcemia, elevated 1,25D levels, PTH suppression, and other evidence of impaired systemic Cyp24a1 effects and excessive systemic 1,25D effects. (**D**) *Villin^CreERT2^Cyp24^fl/fl^* mice show PTH suppression and increased urinary calcium excretion (green line) compared with controls (black) despite no change in circulating levels of calcium or 1,25D. *n* ≥ 4 for all measurements. Results are mean ± SEM. Statistical significance was calculated by mixed effect analysis with Holm-Šidák’s correction and 2-tailed Student’s *t* test. Linear regression was used to analyze the effects of genotype on the relationship between urine calcium standardized to urine creatinine and serum calcium. ^*^P < 0.05; ^**^P < 0.005; ^***^P < 0.0005; ^****^P < 0.0001.

To accentuate the phenotype of tissue-specific *Cyp24a1* deletion, we exposed mice to a higher 2.0% calcium diet for 12 weeks (Figure 6A). High calcium diet precipitated hypercalcemia in *Ubc^CreERT2^Cyp24^fl/fl^* mice (Figure 6B) and exacerbated hypercalcemia in *Six2^Cre^Cyp24^fl/fl^* mice compared with controls (Figure 6C) and compared with experimental mice fed the normal calcium diet (Figure 4, B and C). In contrast, serum calcium remained unchanged in *Villin^CreERT2^Cyp24^fl/fl^* mice, even in response to a high calcium diet (Figure 6D). Interestingly, serum phosphate levels decreased in *Ubc^CreERT2^Cyp24^fl/fl^* mice but increased in *Six2^Cre^Cyp24^fl/fl^* mice; phosphate was unchanged in *Villin^CreERT2^Cyp24^fl/fl^* mice. *Six2^Cre^Cyp24^fl/fl^* mice again demonstrated elevated BUN but no histological evidence of kidney damage (Supplemental Figure 2B). There was no change in BUN in *Ubc^CreERT2^Cyp24^fl/fl^* mice (Figure 6B).

PTH was suppressed and systemic 1,25D, 25D, and FGF23 levels were increased in *Ubc^CreERT2^Cyp24^fl/fl^* and *Six^Cre^Cyp24^fl/fl^* mice fed a high calcium diet (Figure 6, B and C). Despite their unchanged serum calcium, phosphate, 1,25D, and 25D levels, PTH was significantly lower in *Villin^CreERT2^Cyp24^fl/fl^* mice compared with controls (Figure 6D). Urinary calcium excretion was significantly increased in *Villin^CreERT2^Cyp24^fl/fl^* mice, suggesting that increased dietary calcium absorption was the mechanism of PTH suppression; for any given serum calcium, the urine calcium to creatinine ratio was 0.72 units (95% CI 0.18, 1.26; *P* = 0.01) higher in *Villin^CreERT2^Cyp24^fl/fl^* versus control mice (Figure 6D). FGF23 was also elevated in *Villin^CreERT2^Cyp24^fl/fl^* mice, but to a far lesser extent than in the *Ubc^CreERT2^Cyp24^fl/fl^* and *Six^Cre^Cyp24^fl/fl^* mice (Figure 6, B–D). Together, these data indicate that deletion of *Cyp24a1* from the intestine is sufficient to augment local 1,25D effects and suppress PTH without precipitating hypercalcemia or altering systemic vitamin D levels.

### Intestinal Cyp24a1 deletion mitigates secondary hyperparathyroidism in a mouse model of CKD.

**Figure 7:**
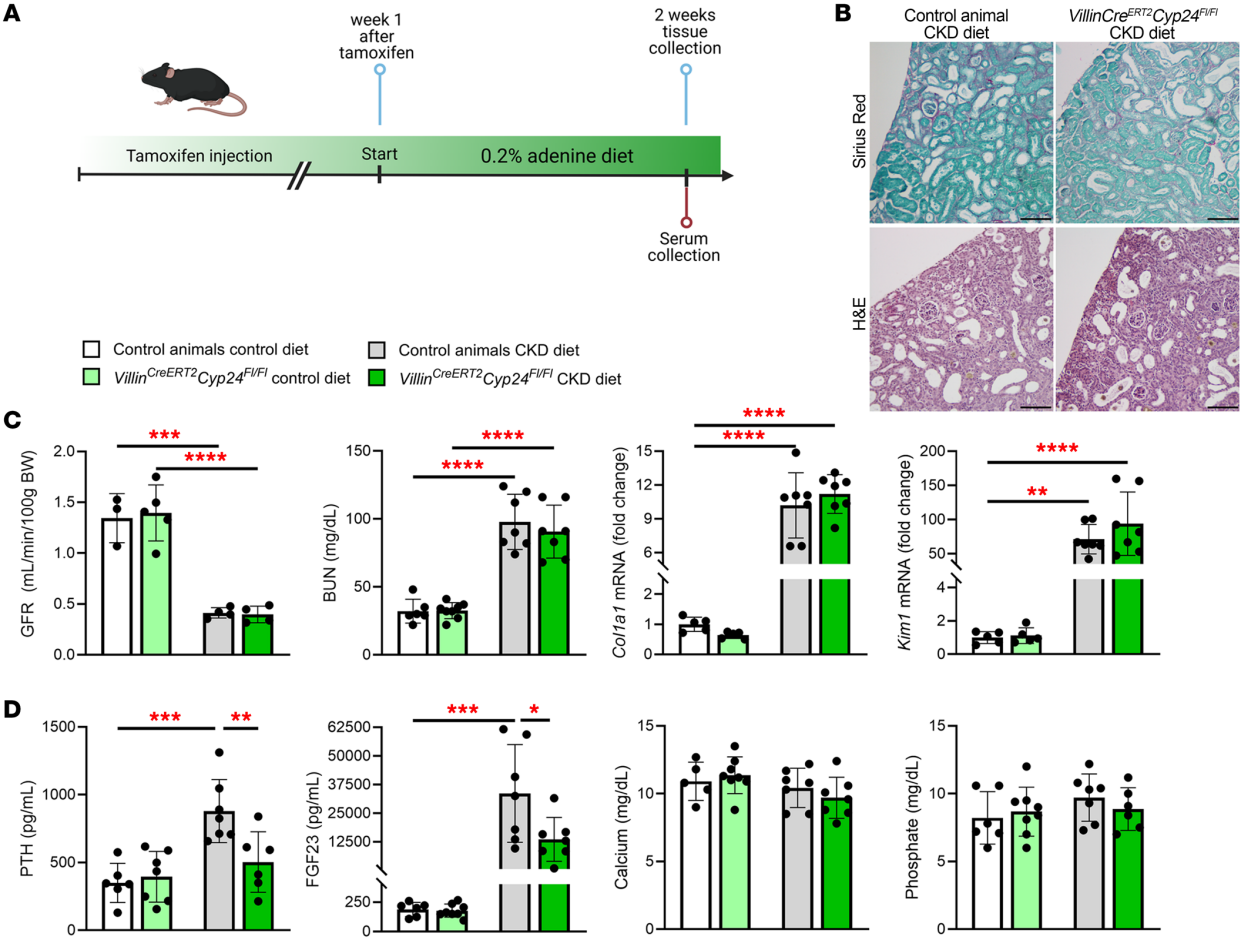
Intestinal deletion of *Cyp24a1* mitigates secondary hyperparathyroidism in a mouse model of kidney damage. (**A**) Kidney damage was induced in male *Villin^CreERT2^Cyp24^fl/fl^* mice and their Cre-negative littermates 1 week after the last tamoxifen injection by feeding them a 0.2% adenine containing diet supplemented with 1% calcium for 2 weeks (CKD diet). Additional *Villin^CreERT2^Cyp24^fl/fl^* and control mice were maintained on the same diet that lacked adenine (control diet). (**B**) Histological examination of kidney tissue indicated a similar level of kidney damage in *Villin^CreERT2^Cyp24^fl/fl^* mice and controls fed the CKD diet. (**C**) Measurement of glomerular filtration rate (GFR), BUN, and the qPCR markers of kidney fibrosis, *Col1a1*, and tubular injury, *Kim1*, confirmed kidney injury in all mice fed the CKD diet, but did not differ between *Villin^CreERT2^Cyp24^fl/fl^* mice and their controls on the CKD diet. (**D**) Compared with controls, *Villin^CreERT2^Cyp24^fl/fl^* mice demonstrate significantly lower PTH and FGF23 levels and unchanged serum calcium and phosphate after 2 weeks on the CKD diet. *n* ≥ 4 for all measurements. Results are mean ± SEM. Statistical significance was calculated by mixed effect analysis with Holm-Šidák’s correction. ^*^P < 0.05; ^**^P < 0.005; ^****^P < 0.0001. Representative images from ≥ 3 animals evaluated for each group. Scale bars: 100μm in **B**.

Secondary hyperparathyroidism is a ubiquitous complication of CKD that increases risks of fracture and cardiovascular disease (1, 5–7). We hypothesized that reducing *Cyp24a1* activity could augment intestinal calcium absorption and mitigate PTH elevation in CKD. Preliminary experiments in *Six2^Cre^Cyp24^fl/fl^* mice demonstrated exacerbation of kidney disease and increased mortality, presumably due to severe hypercalcemia (data not shown). Given the excessive toxicity related to reducing kidney *Cyp24a1* activity in a mouse model of CKD, we fed *Villin^CreERT2^Cyp24^fl/fl^* mice and their Cre-negative controls a diet containing 0.2% adenine supplemented with 1% calcium and no vitamin D for 2 weeks to induce kidney damage (CKD diet, Figure 7A). As an additional control, a second group of *Villin^CreERT2^Cyp24^fl/fl^* and control mice was placed on an identical control diet that lacked adenine.

Histological evaluation of the kidney and measurement of glomerular filtration rate (GFR), BUN, and mRNA markers for kidney fibrosis and tubular damage confirmed significant kidney injury after 2 weeks of the CKD versus the control diet, but there were no differences in these parameters between *Villin^CreERT2^Cyp24^fl/fl^* and control animals fed the CKD diet (Figure 7, B and C). Consistent with the pattern observed in human CKD, PTH and FGF23 levels increased significantly in control animals fed the CKD diet (Figure 7D). In contrast, the increases in PTH and FGF23 levels were significantly attenuated in mice with intestinal deletion of *Cyp24a1* fed the CKD diet despite no changes in serum calcium or phosphate (Figure 7D). These data suggest that intestine-specific deletion of *Cyp24a1* can lower PTH and FGF23 levels in a model of CKD without precipitating hypercalcemia or hyperphosphatemia and without altering the severity of kidney disease.

## Discussion

**Figure 8:**
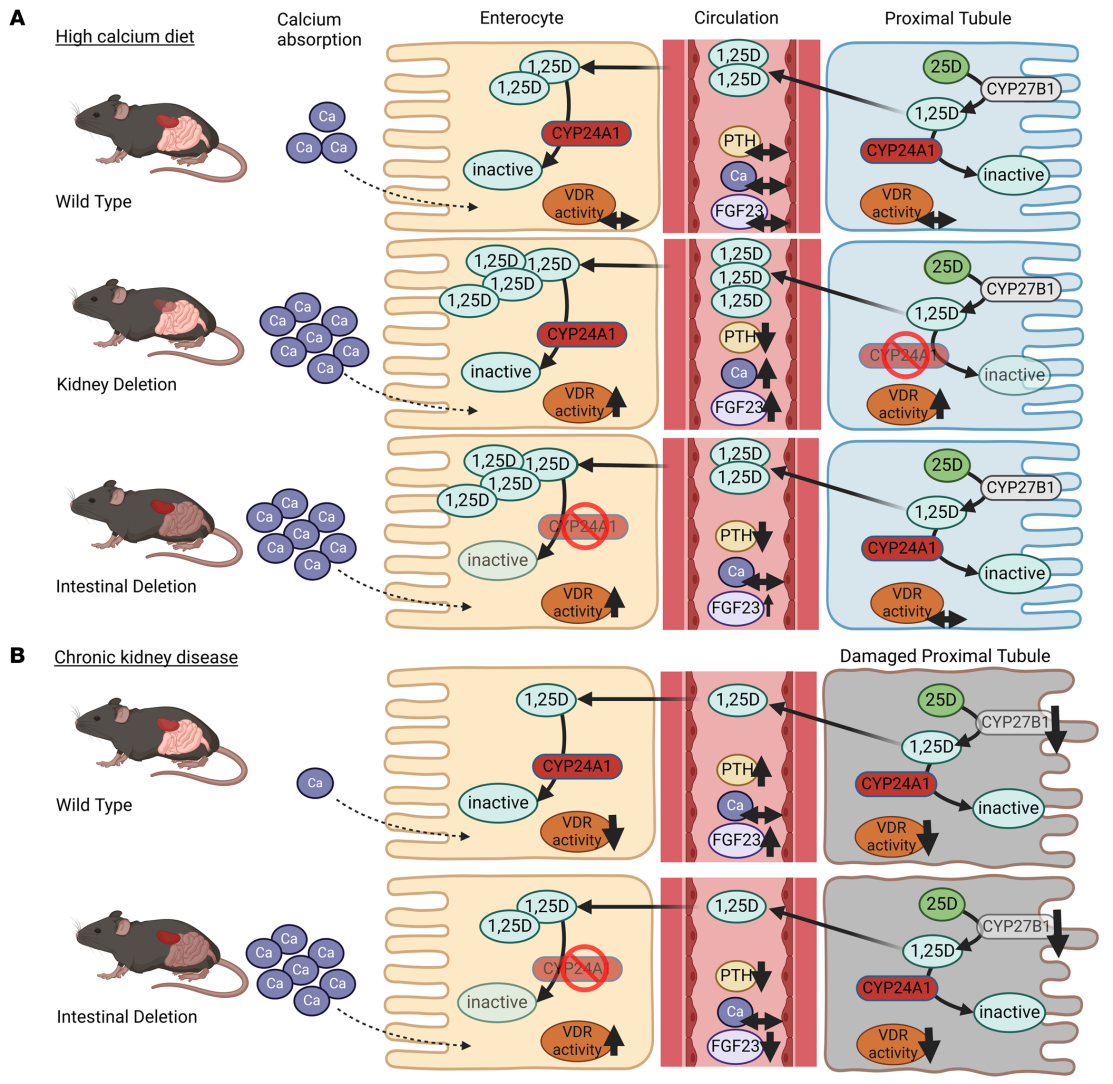
Tissue-specific effects of *Cyp24a1* deletion in health and in CKD. (**A**) Kidney-specific deletion of *Cyp24a1* increased systemic 1,25D levels and 1,25D-VDR activity, which caused hypercalcemia, increased FGF23, and suppressed PTH. In contrast, mice with intestine-specific deletion of *Cyp24a1* demonstrated no change in circulating 1,25D levels, yet 1,25D-VDR activity increased locally in the intestines. As a result, augmented intestinal calcium absorption suppressed PTH without causing hypercalcemia and only mildly increased FGF23. (**B**) Secondary hyperparathyroidism is a common complication of CKD that is characterized by low 1,25D effects. In a model of CKD, intestinal *Cyp24a1* deletion potentiated 1,25D effects locally in the intestine, resulting in increased calcium absorption and significantly decreased PTH and FGF23 levels. These findings support intestinal *Cyp24a1* as a potential target for the treatment of secondary hyperparathyroidism in patients with CKD.

Prior research of vitamin D regulation focused primarily on synthesis of 1,25D from 25D by CYP27B1. Far less attention has been dedicated to catabolism of 1,25D by CYP24A1 despite multiple compelling lines of human genetic evidence that point to its critical importance. Here, we investigated tissue-specific effects of CYP24A1 by exposing global, intestine-specific, and kidney-specific mouse models of *Cyp24a1* deletion to dietary calcium challenges and CKD. We uncovered independent systemic and local effects of CYP24A1 on vitamin D and mineral homeostasis (Figure 8A). Furthermore, our finding that intestine-specific *Cyp24a1* deletion can attenuate secondary hyperparathyroidism in an established model of chronic kidney damage suggests that intestinal CYP24A1 could serve as a therapeutic target in CKD and, potentially, other disorders of mineral homeostasis (Figure 8B).

Prior mouse genetic research of Cyp24a1 deficiency deployed a classic constitutive global knock-out system that precluded detailed investigation of tissue-specific functions of CYP24A1. To address this gap, we generated a mouse line that enabled tissue-specific deletion of *Cyp24a1* by flanking exon 5 with loxP sites. Based on in silico modeling of the predicted mutant Cyp24a1 protein structure, we expected that excision of exon 5 would eliminate *Cyp24a1* function. After crossbreeding *Cyp24^fl/fl^* mice with previously established Cre-recombinase models, we confirmed efficient tissue-specific deletion of *Cyp24a1* using in situ hybridization. Functionally, we confirmed that deletion of exon 5 inactivated *Cyp24a1* using a proximal tubule epithelial cell–based assay of 24,25D production in response to 25D administration and by demonstrating that mice with inducible global deletion of *Cyp24a1* were unable to clear exogenously delivered 1,25D, similar to what was observed in the previous constitutive global knock-out model (27, 42).

Using these newly validated mouse models, we found that deletion of *Cyp24a1* from the kidney is sufficient to replicate the effects of global deletion on mineral homeostasis. Mice demonstrated increased systemic 1,25D and 25D levels, decreased 24,25D, increased 1,25D:24,25D ratio, and increased expression of several mRNA markers of VDR activation in both the kidney and intestine. As a result of increased systemic vitamin D effects and established downstream feedback mechanisms, PTH was suppressed and FGF23 levels were markedly elevated. These effects were magnified when mice were exposed to a high versus standard calcium diet. Interestingly, on the standard calcium diet, serum calcium was significantly elevated in *Six2^Cre^Cyp24^fl/fl^* but not in *Ubc^CreERT2^Cyp24^fl/fl^* mice. Although we found no histological evidence of kidney damage in *Six2^Cre^Cyp24^fl/fl^* mice, it is possible that the longer duration of *Cyp24a1* deficiency in these mice with developmental *Cyp24a1* deletion caused subtle reductions in kidney function, marked by modestly increased BUN, that could account for their higher serum calcium compared with *Ubc^CreERT2^Cyp24^fl/fl^* mice. We speculate that the inducible *Ubc^CreERT2^Cyp24^fl/fl^* that we engineered avoided hypercalcemia because their healthy and normal kidney development enabled them to efficiently excrete their excess calcium load in the urine when *Cyp24a1* deficiency was induced in adulthood. Serum phosphate also increased only in *Six2^Cre^Cyp24^fl/fl^* mice fed a high calcium diet, lending further support for some degree of kidney dysfunction in these animals. In contrast, serum phosphate decreased in *Ubc^CreERT2^Cyp24^fl/fl^* mice fed a high calcium diet, consistent with their 1,25D-induced elevation of FGF23 exerting its known phosphaturic effects on the animals’ healthy kidneys.

In contrast to the *Ubc^CreERT2^Cyp24^fl/fl^* and *Six2^Cre^Cyp24^fl/fl^* mice, *Villin^CreERT2^Cyp24^fl/fl^* mice consuming a standard calcium diet demonstrated evidence of upregulation of VDR-dependent target genes only locally in the intestine and no significant changes in circulating markers of vitamin D or mineral homeostasis. When these animals were fed a high calcium diet, however, PTH was suppressed despite no changes in serum 1,25D or calcium levels. This suggests that increased intestinal calcium absorption due to local increases in 1,25D-VDR activity was appropriately sensed by the parathyroid glands and resulted in suppression of PTH. The significant increase in urinary calcium excretion observed in these mice supports this interpretation and helps explain why these animals were resistant to development of hypercalcemia in response to a high calcium diet. In aggregate, these data indicate that intestinal deletion of *Cyp24a1* is sufficient to modulate total body calcium homeostasis by potentiating local 1,25D effects, which augments intestinal calcium absorption. The resultant suppression of PTH occurs independently of changes in systemic 1,25D levels and without causing hypercalcemia.

These results could have therapeutic implications for patients with CKD in whom disordered mineral homeostasis is a pervasive complication (5, 44). Since enterocytes rely heavily on 1,25D to facilitate dietary calcium absorption (12), FGF23-mediated reductions in 1,25D levels, which begin early in the course of CKD, contribute to development of secondary hyperparathyroidism (24, 25, 45, 46). Based on our data from mice fed a high calcium diet, we hypothesized that inhibiting intestinal CYP24A1 could restore 1,25D effects locally in the intestine and thereby attenuate secondary hyperparathyroidism in CKD without precipitating hypercalcemia.

To test this hypothesis, we fed animals with intact *Cyp24a1* an adenine-containing diet for 2 weeks to model CKD-induced secondary hyperparathyroidism. Mice developed histological, serological, and functional evidence of kidney damage, decreased GFR, increased expression of kidney injury and fibrosis markers, and significantly increased levels of PTH and FGF23 compared with animals fed a control diet. In contrast, PTH levels of *Villin^CreERT2^Cyp24^fl/fl^* mice exposed to an adenine diet were significantly lower than control animals fed adenine and were comparable to animals fed the adenine-free diet. Importantly, we confirmed that PTH reduction was achieved in *Villin^CreERT2^Cyp24^fl/fl^* mice despite no differences in their severity of kidney injury relative to control animals fed the adenine diet and without precipitating an increase in serum calcium.

An unexpected finding was that FGF23 levels were also significantly lower in *Villin^CreERT2^Cyp24^fl/fl^* versus control mice with adenine-induced kidney damage. Directionally, this effect was opposite to the modest but significant increase in FGF23 in *Villin^CreERT2^Cyp24^fl/fl^* mice with intact kidneys that were fed high calcium diet. Established stimuli of FGF23 secretion include 1,25D, serum calcium, and PTH (10, 11). While it is possible that reduced PTH underlies the FGF23 reduction observed in *Villin^CreERT2^Cyp24^fl/fl^* mice with kidney damage, PTH was also reduced in *Villin^CreERT2^Cyp24^fl/fl^* mice with intact kidneys in response to a high calcium diet, yet FGF23 was modestly but significantly increased in that setting. While additional investigation is needed to understand the mechanism of FGF23 reduction in *Villin^CreERT2^Cyp24^fl/fl^* mice with kidney damage, these results support inhibition of intestinal CYP24A1 as an approach to mitigate secondary hyperparathyroidism and FGF23 excess in patients with CKD. Given the strong associations of elevated PTH and FGF23 with bone disease, cardiovascular disease, and mortality (1, 5–7), further research should investigate the long-term effects of intestinal *Cyp24a1* deletion on mineral homeostasis, skeletal histomorphometry, arterial calcification, and left ventricular hypertrophy in adenine-induced and other mouse models of CKD. In the interim, our data suggest that intestinal CYP24A1 could be targeted to enable therapeutic modulation of intestinal calcium absorption without major swings in serum calcium. Such an approach could have clinical benefit in common conditions that are marked by disordered calcium homeostasis such as CKD, osteoporosis, and calcium nephrolithiasis.

## Methods

### Sex as a biological variable.

For experiments involving calcitriol injection and standard or high calcium diet, if mice of both sexes were used and no difference was found between sexes, we report these as combined results. Given known sex differences in susceptibility to adenine-induced nephropathy (47, 48), we focused on male mice that develop more severe kidney damage to prove the principal mechanism of our hypothesis.

### Animals.

Mice were maintained in temperature-controlled environments (22–23°C) with 12-hour light-dark cycles. Mice were provided ad libitum access to water and standard rodent diet containing 0.8% calcium, 0.6% phosphorus and 2.3 IU/g cholecalciferol (LabDiet) or 2% calcium, 1.25% phosphorus and 2.2 IU/g cholecalciferol (Envigo, TD.94112). All mice were bred on a C57/Bl6/J background with at least 10 generations of backcrossing. WT mice of both sexes used for this study were procured from Jackson Laboratories and analyzed at about 12 weeks of age (Jackson Laboratories No. 000664). Mice with floxed alleles for *Cyp24a1* were generated by GeneCopoeia using CRISPR/Cas9 to insert loxP sites flanking exon 5 of *Cyp24a1* (Reference ID: TGM-76991). Mice carrying the *Cyp24a1* floxed allele were bred homozygous as *Cyp24a1^flox/flox^* (abbreviated as *Cyp24^fl/fl^*) and crossed with *Ubc-CreERT2* (*B6.Cg-Ndor1^Tg(UBC–cre/ERT2)1Ejb^/1J* abbreviated as *Ubc^CreERT2^*; Jackson Laboratories No. 007001), *Six2-TGC^tg^* (*Tg(Six2-EGFP/cre)1Amc/J* abbreviated as *Six2^Cre^*; Jackson Laboratories No. 009606), and *Villin-CreERT2* (*B6.Cg-Tg(Vil1-cre/ERT2)23Syr/J* abbreviated as *Villin^CreERT2^*; Jackson Laboratories No. 020282). Organ specificity of *Villin-CreERT2* was confirmed by crossing with a *Rosa26^tdTomato^* reporter strain (*B6.Cg-Gt(ROSA)26Sor^tm14(CAG–tdTomato)Hze^/J* abbreviated as *Rosa26^tdTomato^*; Jackson Laboratories No. 007914). Expression of Cre-recombinase was heterozygous for all experimental mice and Cre-negative littermates served as controls for all experiments. Mice were terminated in the mornings of the final experimental day.

### Tamoxifen injection to induce Cre-recombinase.

All *Ubc^CreERT2^* and *Villin^CreERT2^* mice were treated with tamoxifen (Merck) dissolved in corn oil (Sigma Aldrich) and ethanol to a final concentration of 20 mg/mL and injected i.p. after disinfecting the skin with 70% ethanol at 12 weeks of age. Induction of Cre-recombinase was performed as previously reported (40, 49) with slight modifications to dosage and frequency of injections, which were tested in preliminary experiments to optimize efficiency. All experimental interventions started a week after the last injection. Cre-negative littermates received the same treatment.

### Calcitriol injections.

Calcitriol (Sigma Aldrich) was dissolved in ethanol and corn oil to a concentration of 0.5 μg/mL. Mice received bodyweight-adjusted doses of calcitriol or corn oil via i.p. injection.

### Serum chemistry.

Blood was collected from the submandibular vein and at study termination via cardiac puncture into Microvette heparin plasma tubes (Sarstedt) and centrifuged at 21,000*g* for 10 minutes. Plasma supernatants were snap frozen in liquid nitrogen and stored at –80°C until analysis. Calcium, phosphate, creatinine, and BUN were measured at the University of North Carolina Animal Clinical Laboratory Services Core with a Vet Axcel Chemistry Analyzer (Alfa Wassermann Diagnostic Technologies). Intact FGF23 and PTH levels were determined by ELISA (QuidelOrtho) according to the manufacturer’s protocol. Levels of 1,25D, 25D, and 24,25D were measured by the Department of Laboratory Medicine and Pathology, at the University of Washington by immune-extraction combined with LC-MS.

### Adenine diet.

To induce experimental kidney damage, male mice were fed a casein-based diet containing 0.2% adenine, 1% calcium, 0.9% phosphorus, and no added vitamin D (Envigo, TD.230742) for 2 weeks. Additional mice were maintained on a control diet of the same composition, but lacking adenine (Envigo, TD.230741). Experimental diets were initiated a week after the last tamoxifen injection. Mice were monitored for weight, food intake, and overall health throughout the experimental period.

### Glomerular filtration rate.

Glomerular filtration rate was determined as previously described by transdermal measurement of fluorescein isothiocyanate-labeled sinistrin clearance (50, 51). Briefly, mice were prepared by shaving and depilation 1 day before the measurement. On the last experimental day mice were anesthetized with isoflurane before having a miniaturized fluorescence detector (MediBeacon) applied using double-sided adhesive tape. The background signal was recorded for 3 minutes before i.v. injection of 12.5 mg/kg fluorescein isothiocyanate-labeled sinistrin (MediBeacon). Animals were returned to consciousness in a single-house cage, and measurements were taken for approximately 1 hour before the device was removed. Data were analyzed using MB Lab/MB Studio software (MediBeacon), with GFR (mL/min/100 g body weight) calculated from the decrease of fluorescence intensity over time using a 3-compartment model and animals’ body weight (51, 52).

### Tissue fixation.

Adult mice were anesthetized by isoflurane inhalation anesthesia, euthanized by exsanguination, and vital organs were removed. To limit the number of required mice, right kidneys were used for mRNA measurements and left kidneys were halved and fixed either in 10% neutral buffered formalin (NBF) solution for in situ hybridization or 4% buffered paraformaldehyde solution for histology. Intestinal samples were fixed in 10% NBF and embedded in paraffin in parallel to kidney tissue. Tissue was fixed overnight and embedded by the Duke Substrate Services Core Research Support.

### Isolation of intestinal epithelial cells.

Upon study termination, the duodenum was cleaned by repeated flushing with ice cold PBS (Gibco) and placed in PBS on ice. PBS was removed and replaced with cold EDTA (25mM). After vigorous shaking, the supernatant was removed to a fresh 50 mL tube and placed on ice. Extraction of cells was repeated by adding fresh EDTA. Tissue was shaken again, supernatants combined, and residual tissue discarded. Supernatant was centrifuged for 3 minutes at 800*g*. After discarding the supernatant, the pellet was washed with fresh PBS. After centrifugation for 5 minutes at 21,000*g*, supernatant was removed, and cells were snap frozen in liquid nitrogen and stored at –80°C until mRNA isolation.

### RNA isolation and quantification.

Total RNA was extracted from kidney and intestinal epithelial cells using a RNeasy Plus Mini Kit (Qiagen) following the manufacturer’s instructions. For purification of intestinal mRNA, RNase-Free DNase Set (Qiagen) was applied according to the manufacturer’s protocol. After isolation, 1 μg of RNA was reverse transcribed to cDNA using Applied Biosystems High-Capacity cDNA Reverse Transcription Kit (cat. 4368813). Quantitative real-time PCR was performed with SSoAdvanced Universal Probe Supermix (Biorad) and sequence-specific TaqMan probes (Thermo Fisher Scientific; Supplemental Table 2). Samples were run in duplicate on a Quantstudio 3 real time detection instrument (Applied Biosystems). Gene expression was normalized to expression levels of β2-microglobulin. Results were evaluated using the 2−ΔΔCt method and expressed as mean ± SEM.

### RNAscope and basescope in situ hybridization.

Localization of mRNA expression was studied with the RNAscope Multiplex Fluorescent v2 kit (Advanced Cell Diagnostics) according to the manufacturer’s instructions and as described previously (53). Basescope was performed with the BaseScope Reagent Kit v2-RED according to the manufacturer’s protocol (Advanced Cell Diagnostics). A Basescope probe specific for exon 5 of *Cyp24a1* was produced by Advanced Cell Diagnostics. RNAscope and BaseScope probes are listed in Supplemental Table 3.

### In silico modelling of protein structure of Cyp24a1.

To predict possible effects of exon 5 deletion on the downstream amino acid sequence of Cyp24a1, we utilized EnzymeX software (version 3.3.3) (54). We performed 3D modeling of mouse WT and mutant Cyp24a1 after deletion of exon 5 with the I-TASSER online resource (55) and compared the resulting models to the previously published crystal structure of rat Cyp24a1 (34). We visualized protein structures from the in silico modeling using Pymol (version 2.6.0) (56).

### In vitro system of primary proximal tubule cell culture to assess Cyp24a1 function.

We isolated proximal tubule cells from kidneys of *Six2^Cre^Cyp24^fl/fl^* mice and their respective controls, as described previously (57). Briefly, kidneys were decapsulated and the medulla was removed, followed by a wash step in cold PBS. Tissue was minced and incubated for 30 minutes at 37°C in digestion buffer (Collagenase type IV Thermo Fisher Scientific 17104019; 1 mg/mL in HBSS). Digestion was stopped with 10% FBS, (USDA Approved, cat. No 35-010-CV, Corning) in PBS. Glomeruli and tissue clumps were allowed to separate for 2 minutes by gravity sedimentation. The top layer of cell suspensions was collected and run through a 70 μm filter into a new tube. Suspension was centrifuged (300*g*, 4°C, 5 minutes) and supernatant discarded. The pellet was washed with PBS. Cells were resuspended with culture medium (DMEM:F12; cat. 10-013-CV, Corning; supplemented with FBS; Pen/Strep, cat. 45000-652, VWR-Corning; ITS, cat. 41400-045, Gibco; Hydrocortisone, cat. H0135, Sigma Aldrich; EGF, cat. E4127, Sigma Aldrich; vitamin C, cat. A4403, Sigma Aldrich) and seeded into 6-well plates. Cells were grown to just before confluency and treated with either vehicle (fresh culture medium) or 25D, 7 nM (Cayman chemical cat. 84988, dissolved in EtOH 1 mg/mL), diluted with culture medium for 12 hours. Culture medium was removed and frozen at –80°C until analysis.

### Single cell preparation of mouse intestine and kidney.

Duodenum fragments were dissociated by enzymatic and mechanical digestions (see Supplemental Methods). To gain equally distributed cell types from crypts and villi, we separated crypts and villi with 10 mM EDTA-PBS digestion, followed by size separation with a 70 μm cell strainer. Single-cell suspension from each fraction was separately prepared using TrypLE Express (Gibco, Ref: 12605-010) for crypts, and collagenase type 1 (6900 U/mL; Worthington, cat. LS004196) for villi. Dead cells were removed using a dead cell removal kit (Miltenyi Biotec cat. 130-090-101). Crypt and villi cells were mixed at a 1:1 ratio, and the final cell suspension was used for library generation. The kidney single-cell suspension was generated as previously reported (58). Briefly, the kidneys were dissociated with liberase TM, hyaluronidase, DNase I for 20 minutes, followed by 0.25% trypsin EDTA with DNase I (20 μg/mL) for 10 minutes. The digestion protocol yielded high cell viability (> 90%) and very few doublets (59). The samples were targeted to 10,000 cell recovery and processed using 10x Chromium Single Cell 30 Reagent kit v3.1 (10x Genomics). cDNA libraries were sequenced using HiSeq X Ten with 150-bp paired-end sequencing. Each condition used cells from 3 mice to reduce biological and technical variability.

### Analyses of scRNA-seq datasets.

Analyses of scRNA-seq data were performed as previously described (58, 59) by processing FASTQ files using 10x Genomics Cell Ranger; reads were mapped on the mm10 mouse genome reference. Unique molecular identifier counts were analyzed using R package Seurat v.4.2.0 (60). We used strict quality control to remove low quality and doublet cells with custom cutoff (duodenum: genes expressed in > 1 cells, cells expressing more than 500 and cells with % mitochondrial genes < 0.25 were included; kidneys: genes expressed in > 1 cell, cells expressing 200–7,500 detected genes, and cells with % mitochondrial genes < 0.60 were included). SoupX and DoubletFinder were used (61, 62). Harmony was used to correct potential batch effects and integrate count matrices from each sample (63, 64). Cluster-defining markers were used to assign a cell identity to each cluster (59, 65). See Supplemental Methods for further details.

### Microscopy.

All micrographs were captured with an Axio Observer.Z1 Microscope (Zeiss), a Plan-Apochromat ×20/0.8 objective, a ×1 tube lens, and the Apotome.2 system (Zeiss), as described previously (53) at the Duke Light Microscopy Core Facility. Images represented in the same figure were always captured with the same light intensities and exposure times.

### Histology.

Sirius Red staining was performed as previously described (66). Sections were mounted with Cytoseal 60 mounting medium (Thermo Fisher Scientific). For H&E staining, slides were incubated in hematoxylin solution (VWR), washed,and placed in 0.5% eosin solution (VWR) followed by 2 washing steps. Sections were dehydrated and mounted as described for Sirius red staining. Tissue from different genotypes for the same intervention were always stained in the same batch and white balance was set according to the control image equally for all images in the set.

### Statistics.

Data are presented as means ± SEM. Identification of possible statistical outliers was performed by ROUT method (*Q* = 1%) in GraphPad Prism. Paired comparisons were analyzed by 2-tailed *t* test. Multiple comparisons were evaluated by mixed effect analysis with Holm-Šidák’s correction for multiple comparisons. Linear regression was used to analyze the effects of genotype on the relationship between urine calcium standardized to urine creatinine and serum calcium. *P* < 0.05 was considered statistically significant. Data were analyzed using GraphPad Prism9 (Graphpad Software) and Stata SE18 (StataCorp).

### Study approval.

All mouse experiments were conducted according to the NIH *Guide for the Care and Use of Animals in Research* and were approved by the Institutional Animal Care and Use Committees at the Duke University School of Medicine, protocol number A238-20-12 and A229-23-11.

### Graphical design.

Experimental timelines and schematics were generated with Biorender. Licenses for illustration: Fuchs, M. (2024) https://BioRender.com/s74n664

### Data availability.

All data used in the figures are represented in the Supporting Data Values file available online. Raw data of the scRNA-seq analysis performed for this study are available at the Gene Expression Omnibus database under accession number GSE254568.

## Author contributions

Research approach was designed by AG, JR, TS, and MW. Acquisition of data and experiments was performed by MAAF, AG, MS, SLM, EJB, NL, VT, SI, KA, and HK. Data were analyzed by MAAF, AG, MS, SLM, and MW, and the manuscript draft was written by MAAF and MW. The final manuscript underwent critical review and editing by MAAF, AG, MS, SLM, EJB, NL, VT, JR, SI, KA, HK, TS, and MW. Regarding the 2 cofirst authors on this manuscript, the senior author assigned the postdoctoral fellow who conducted most of the experiments and led the drafting of the manuscript text and figures to be listed first among the 2 cofirst authors and the supervising scientist to be listed second.

## Supplementary Material

Supplemental data

Supporting data values

## Version 1 12/17/2024

In-Press Preview

## Version 2 02/17/2025

Electronic publication
