# Supplementary material for: Intestinal Cyp24a1 regulates vitamin D locally independent of systemic regulation by renal Cyp24a1 in mice
Source: J Clin Invest. 2024 Dec 17;135(4):e179882. doi: 10.1172/JCI179882 (PMC11827884; doi:10.1172/JCI179882)
Supplement: Supplemental data [file jci-135-179882-s144.pdf]

Supplement to:

Intestinal *Cyp24a1* regulates vitamin D locally independent of systemic regulation by renal *Cyp24a1* in mice

Michaela A.A. Fuchs[1*], Alexander Grabner[1,2,3*], Melody Shi[1], Susan L. Murray[1], Emily J. Burke[1], Nejla Latic[4], Venkataramana Thiriveedi[5], Jatin Roper[5], Shintaro Ide[1], Koki Abe[1], Hiroki Kitai[1], Tomokazu Souma[1], Myles Wolf[1,6,7]

* Co-first authors

Contents:

- Supplemental Figures: 2
- Supplemental Tables: 3
- Supplemental Methods
- Supplemental References

Supplemental Figures and Legends

Supplemental Figure 1. Cell-type assignment for scRNAseq analysis and *Vdr* expression in kidney and small intestines.

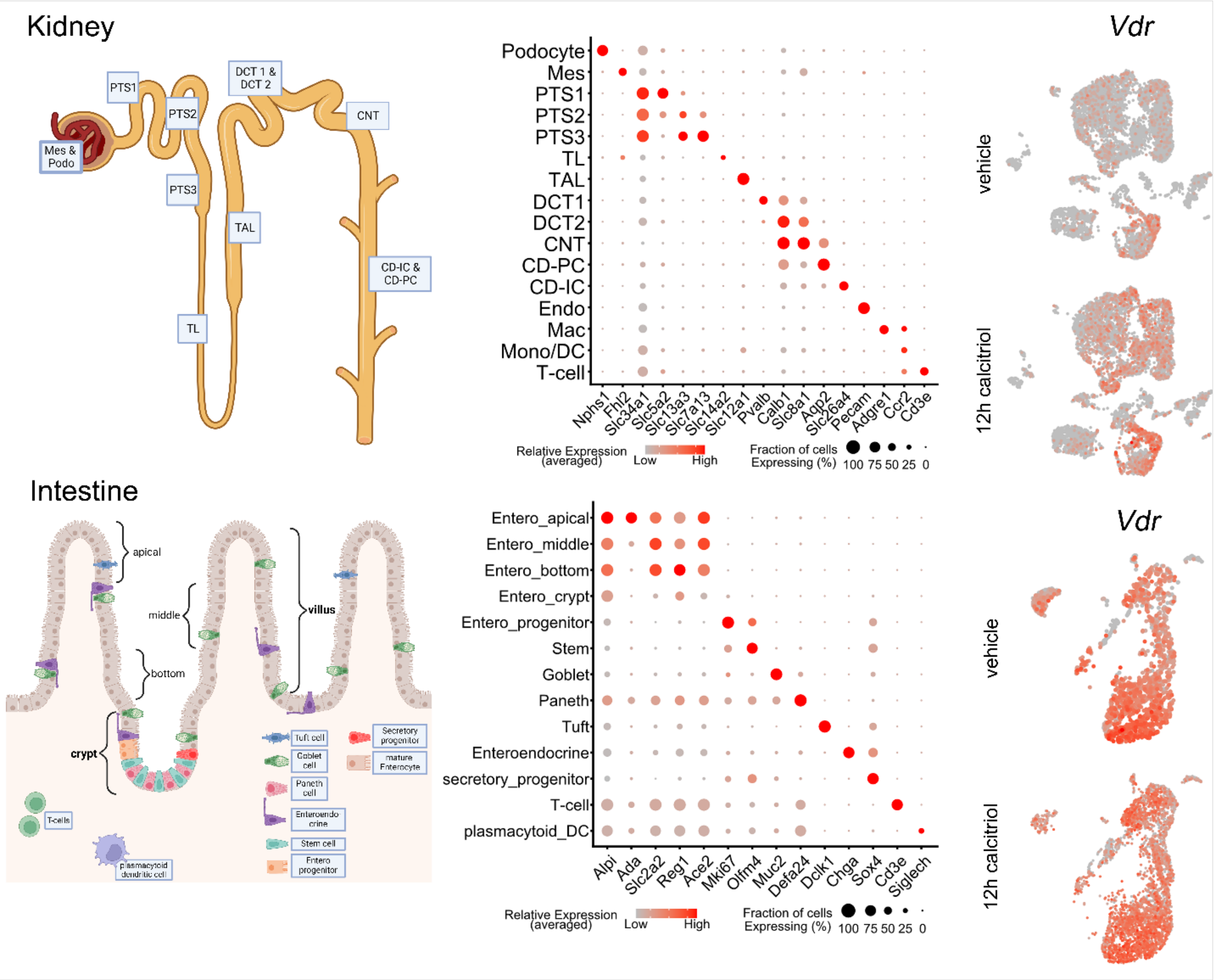

Schema indicates the cell types analyzed by scRNAseq in the kidney (top) and duodenum (bottom). Dot plot shows expression pattern of cluster-enriched marker genes. *Vdr* was expressed in most cell types of the kidney and showed a modest increase 12 hours after calcitriol injection. In the intestine, *Vdr* was widely expressed at baseline and did not show major changes after calcitriol treatment. Abbreviations: PT, proximal tubule (S1, S2, S3 segments); TL, thin limb; TAL, thick ascending limb; DCT, distal convoluted tubule; CNT, connecting tubule; CD, collecting duct; PC,

principal cells; IC, intercalated cells; Mes, mesangial cells; Endo, endothelial cells; Mac, macrophages; Mono/DC, monocytes and dendritic cells; Neut, neutrophils: Entero, enterocyte; Stem, stem cell.

Supplemental Figure 2. Kidney histology of mice with tissue-specific deletion of *Cyp24a1*.

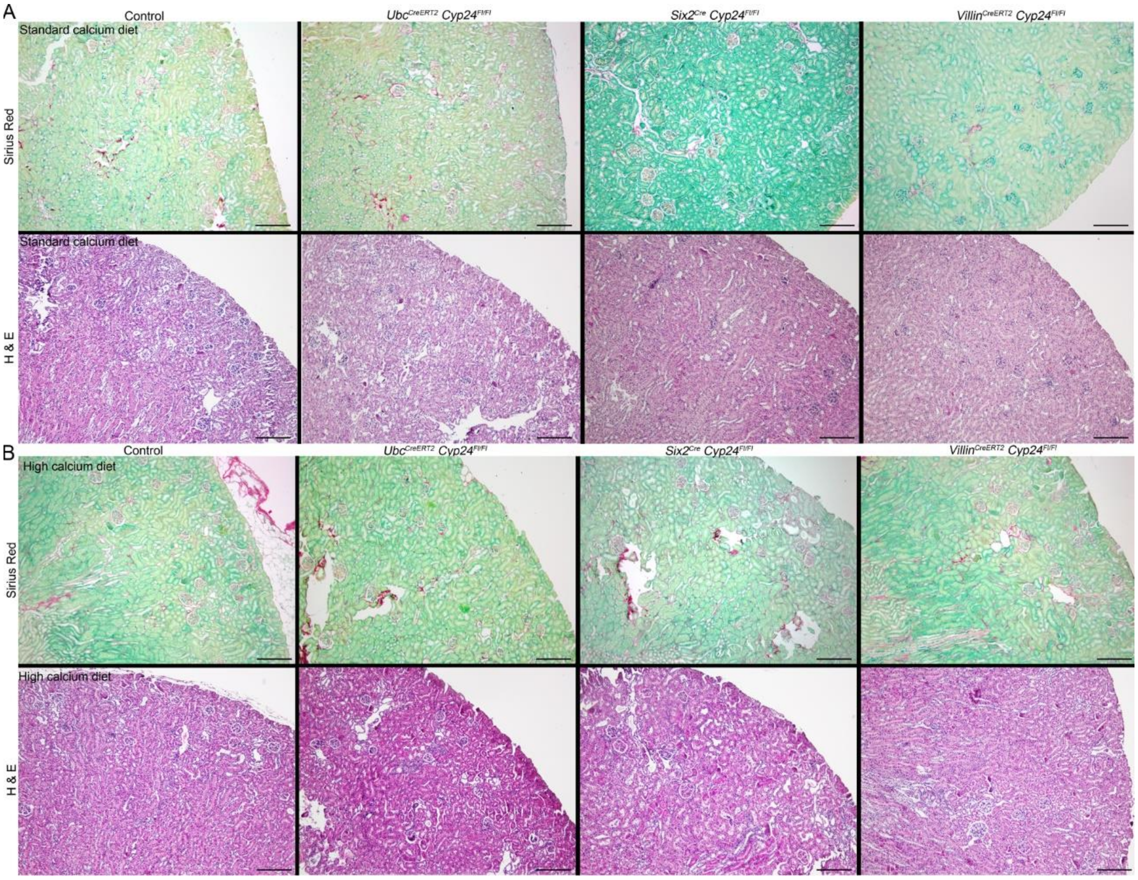

Sirius red and hematoxylin and eosin (H&E) staining of kidney tissue demonstrate no histological evidence of kidney damage in *Ubc*^{CreERT2}*Cyp24*$^{Fl/Fl}$, *Six2*^{Cre}*Cyp24*$^{Fl/Fl}$ and *Villin*^{CreERT2}*Cyp24*$^{Fl/Fl}$ mice in response to (A) standard calcium diet, or (B) high calcium diet. Representative images from the ≥ 4 animals of each strain that were evaluated. Scale bars, 200 µm.

Supplemental Tables

Supplemental Table 1. Functionally relevant amino acid residues of CYP24A1.

Residue and position in wildtype CYP24A1	Characteristic of residue	Mutant 1	Mutant 2
**W75**	Functionally important	+	-
**Y89**	Functionally important	+	-
**L129**	Functionally important	+	-
**I131**	Functionally important	+	-
**W124**	Functionally important	+	-
**E143**	Pathological SNP	+	-
**R159**	Pathological SNP	+	-
**L236**	Functionally important	+	-
**M245**	Functionally important	**G**	+
**M246**	Functionally important	**H**	+
**F249**	Functionally important	**Q**	+
**H271**	Functionally important	**L**	+
**W275**	Functionally important	-	+
**L325**	Functionally important	-	+
**A326**	Functionally important	-	+
**E329**	Functionally important	-	+
**E322**	Pathological SNP	-	+
**T330**	Functionally important	-	+
**K378**	Functionally important	-	+
**K382**	Functionally important	-	+
**V391**	Functionally important	-	+
**F393**	Functionally important	-	+
**T394**	Functionally important	-	+
**T395**	Functionally important	-	+
**R396**	Pathological SNP	-	+
**L409**	Pathological SNP	-	+
**R465**	Functionally important	-	+
**R466**	Functionally important	-	+
**G499**	Functionally important	-	+

Amino acid residues with high relevance for enzyme function, protein morphology and catalytic activity as well as selected human pathological SNPs were included in the analysis of possible mutant proteins that could result from the deletion of exon 5. Only residues that were conserved between the human and wild-type sequence of Cyp24a1 were included for analysis. + = residue is conserved, - = residue is lost due to structural changes, letters represent changed amino acids due to frame shift.

Supplemental Table 2. TaqMan probes used for mRNA quantification.

Target	Assay ID
***B2m*, encoding Beta-2 microglobulin**	Mm00437762_m1
***Cyp24a1* (targeting exon 5 and 6)**	Mm01252894_m1
Cyp27b1	Mm01165918_g1
Trpv5	Mm01166037_m1
Trpv6	Mm00499069_m1
S100g	Mm00486654_m1
Col1a1	Mm00801666_g1
Havcr1 (Kim1)	Mm00506686_m1

TaqMan probes used for quantitative mRNA analysis. All presented values were normalized to the expression of Beta-2 microglobulin.

Supplemental Table 3. Probes used for RNAscope or BaseScope

Target	Probe catalog number	Assay
Cyp24a1	448401	RNAscope
***Cyp24a1* exon 5 only**	1264881-C1	BaseScope

Probes used for in situ hybridization, all probes were manufactured by Advanced Cell Diagnostics.

Supplemental Methods

Single cell preparation of mouse small intestine

Washed duodenum fragments, 2 cm in length, were incubated in 10 mM EDTA-PBS for 20 minutes at 4°C, and then washed in PBS with gentle shaking. Next, cell suspensions were filtered through a 70-µm cell strainer to separate crypt and villous fractions (crypt: <70 µm; villi, >70 µm). Crypts fractions were further digested with TrypLE Express (Gibco, Ref: 12605-010), and villi fractions with collagenase type 1 (6900 U/mL; Worthington, cat. No. LS004196) at 37°C for 1 min 15 seconds. S-MEM medium (Gibco, Ref:11380-037) was added to stop the digestion reactions. The single cells were resuspended and filtered through a 40-µm cell strainer with added mechanical agitation. Dead cells were removed from the suspension using a dead-cell removal kit (Miltenyi Biotec cat. No.130-090-101). The crypt and villi cell fractions were mixed at a 1:1 ratio and the final cell suspension was used for library generation. We confirmed high cell viability and efficient dissociation with trypan blue staining prior to library generation (viability 95%).

Single cell preparation of mouse kidneys

The kidneys were dissociated with liberase TM, (0.3 mg/mL, Roche, cat. No. 291963), hyaluronidase (10 µg/mL, Sigma, H4272) and DNase I (20 µg/mL) at 37°C for 20 min, followed by incubation with 0.25% trypsin EDTA with DNase I (20 µg/mL) at 37°C for 10 min. Trypsin was inactivated using 10% fetal bovine serum in PBS. Cells were then resuspended in PBS supplemented with 0.04% bovine serum albumin. Cell suspensions obtained after filtration through a 40-µm strainer were used for library generation. Our protocol yielded high cell viability (>90%) and very few doublets, as previously reported (2).

scRNAseq library generation

Kidney and intestinal cells were processed at the Duke Human Vaccine Institute for library generation. The samples were targeted to 10,000 cell recovery and processed using 10x Chromium Single Cell 30 Reagent kit v3.1 (10x Genomics). cDNA libraries were sequenced using HiSeq X Ten with 150-bp paired-end sequencing. Each condition analyzed (vehicle and 12h calcitriol injection) contains cells from 3 mice to minimize potential biological and technical variability.

Data preprocessing, unsupervised clustering, and cell type annotation

Analysis of the raw scRNAseq data was performed by processing FASTQ files using 10x Genomics Cell Ranger. Reads were mapped on the mm10 mouse genome reference. Background signals were filtered out using SoupX (ver.1.6.2) (3). Unique molecular identifier counts were analyzed using R package Seurat v.4.2.0 for quality control, dimensionality reduction, and cell clustering (4). Low quality cells were identified and removed prior to further analysis using custom cutoff settings (duodenum: genes expressed in >1 cells, cells expressing more than 500 and cells with % mitochondrial genes < 0.25 were included; kidneys: genes expressed in >1 cell, cells expressing 200-7,500 detected genes, and cells with % mitochondrial genes < 0.60 were included). DoubletFinder (ver. 2.03) was used to identify and remove potential doublets (5). We then used Harmony (ver.1.0.3) to correct for potential batch effects and integrate count matrices from each sample (6, 7). To further limit confounding, the mitochondrial mapping percentage was regressed out. The resulting integrated dataset was used for all downstream analyses. A graph-based clustering approach in Seurat was used to cluster cells, and the resolution was set at 1.2. Cluster-defining markers for each cluster obtained using the Seurat's FindAllMarkers command (genes expressed by at least 20% of cells within the cluster, log fold change > 0.25) with the Wilcoxon Rank-Sum test. Based on the marker genes and manual curation of the gene expression pattern of

canonical marker genes in UMAP plots, we assigned a cell identity to each cluster (2, 8). Secretory progenitor cells were identified by the high expression of Sox4 (9). Villous enterocytes express unique genes based on the location (Top, middle, and bottom) of the villi; these were used to distinguish enterocytes into distinct clusters (10). *Ace2* is highly expressed in mature enterocytes of villi (11) and *Olfm4* is highly expressed in intestinal stem cells. Progenitors of enterocytes (Entero_Progenitors) were clustered by the simultaneous expression of stem cell markers and key markers for entry into the cell cycle (MKi67). We excluded ambiguous clusters with mixed identities from downstream analyses of the intestinal datasets as they most likely represented doublets.

Supplemental References

1. Annalora AJ, et al. The crystal structure of CYP24A1, a mitochondrial cytochrome P450 involved in vitamin D metabolism. *J Mol Biol*. 2010;396(2):441–451.

2. Ide S, et al. Ferroptotic stress promotes the accumulation of pro-inflammatory proximal tubular cells in maladaptive renal repair. *eLife*. 2021;10:e68603.

3. Young MD, Behjati S. SoupX removes ambient RNA contamination from droplet-based single-cell RNA sequencing data. *Gigascience*. 2020;9(12):giaa151.

4. Hao Y, et al. Integrated analysis of multimodal single-cell data. *Cell*. 2021;184(13):3573-3587.e29.

5. McGinnis CS, et al. DoubletFinder: Doublet detection in single-cell rna sequencing data using artificial nearest neighbors. *Cell Syst*. 2019;8(4):329-337.e4.

6. Korsunsky I, et al. Fast, sensitive and accurate integration of single-cell data with Harmony. *Nat Methods*. 2019;16(12):1289–1296.

7. Chazarra-Gil R, et al. Flexible comparison of batch correction methods for single-cell RNA-seq using BatchBench. *Nucleic Acids Res*. 2021;49(7):e42.

8. Haber AL, et al. A single-cell survey of the small intestinal epithelium. *Nature*. 2017;551(7680):333–339.

9. Gracz AD, et al. Sox4 promotes atoh1-independent intestinal secretory differentiation toward tuft and enteroendocrine fates. *Gastroenterology*. 2018;155(5):1508-1523.e10.

10. Moor AE, et al. Spatial Reconstruction of Single Enterocytes Uncovers Broad Zonation along the Intestinal Villus Axis. *Cell*. 2018;175(4):1156-1167.e15.

11. Ohara TE, et al. Adaptive differentiation promotes intestinal villus recovery. *Dev Cell*. 2022;57(2):166-179.e6.
